# Redox‐Active Hybrid Polyoxometalate‐Stabilised Gold Nanoparticles

**DOI:** 10.1002/anie.202005629

**Published:** 2020-06-29

**Authors:** Carmen Martin, Katharina Kastner, Jamie M. Cameron, Elizabeth Hampson, Jesum Alves Fernandes, Emma K. Gibson, Darren A. Walsh, Victor Sans, Graham N. Newton

**Affiliations:** ^1^ Nottingham Applied Materials and Interfaces (NAMI) Group The GSK Carbon Neutral Laboratories for Sustainable Chemistry University of Nottingham Nottingham NG7 2TU UK; ^2^ Universidad de Sevilla Departamento de Quimica Fisica Facultad de Quimica 41012 Sevilla Spain; ^3^ School of Chemistry University of Nottingham University Park Nottingham NG7 2RD UK; ^4^ School of Chemistry University of Glasgow Glasgow G12 8QQ UK; ^5^ Institute of Advanced Materials (INAM) University Jaume I 12006 Castellon Spain

**Keywords:** gold nanoparticles, hybrid materials, photochemistry, polyoxometalates, redox chemistry

## Abstract

We report the design and preparation of multifunctional hybrid nanomaterials through the stabilization of gold nanoparticles with thiol‐functionalised hybrid organic–inorganic polyoxometalates (POMs). The covalent attachment of the hybrid POM forms new nanocomposites that are stable at temperatures and pH values which destroy analogous electrostatically functionalised nanocomposites. Photoelectrochemical analysis revealed the unique photochemical and redox properties of these systems.

## Graphical abstract

**Handy hybrids**: A material based on gold nanoparticles stabilized by organic–inorganic hybrid polyoxometalates is described (see picture). The new nanomaterial exhibits unprecedented stability and photoactivity under visible light and can be reversibly reduced and oxidized at electrode surfaces, making it very promising for future photonic and electrochemical systems.

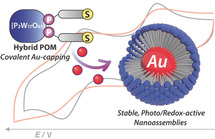

Owing to their unique catalytic, electronic, and optical properties, gold nanoparticles (AuNPs) are used in a wide range of areas, including healthcare, sensing, catalysis, and photonics.^1^ To prevent agglomeration of AuNPs during nucleation and growth, a range of surfactants and capping agents are often used in their synthesis. These capping agents can also be designed to impart added functionality, such as redox activity.^2^ In this regard, the use of redox‐active polyoxometalates (POMs) offers a range of material‐design opportunities owing to their stability and enormous structural and compositional diversity. POMs are anionic metal oxide clusters that exhibit rich electrochemical and photochemical properties,^3^ and are increasingly popular reducing agents^4^ and electrostatic capping agents^5^ for the synthesis of metal NPs and nanocomposites.^6^

In parallel to the development of NP@POM composites, the past decade has seen a rapid increase in the development of organic/inorganic hybrid POMs, in which organic groups are covalently tethered to the metal oxide cluster.^7^ The properties of such materials can be finely tuned by changing the nature of the POM and the organic modifier,^8^ paving the way for the rational molecular design of new multifunctional supramolecular materials.^7^, ^9^ For example, it was recently shown that POMs could be functionalised with organophosphoryl groups to prepare a new family of amphiphilic hybrid POM surfactants.^10^ The surfactant molecules self‐assemble in solution to form micellar aggregates that retain the (modified) redox properties of the molecular building blocks, and similar nanoscale systems have been proposed as charge carriers for use in solution‐phase energy‐storage systems.^11^ Hybrid POMs have also been used as capping agents for AuNPs,^12^ whereby thiol‐terminated organosilane‐functionalised clusters are used to covalently stabilise the NP surface. This strategy is notable in that it minimises desorption of the POM capping groups and presents additional opportunities for synthetic control over the system. For example, Polarz and co‐workers have recently shown how synergistic interactions between appended hybrid POMs and the surface‐plasmon resonance of AuNPs can enhance photocatalytic activity.^13^ Although these studies point to the considerable promise of such materials, our understanding of the stability and emergent properties of these systems remains very much in its infancy, thus limiting our ability to rationally prepare new nanomaterials with properties tailored towards specific, novel applications.

Herein, we describe the synthesis and properties of AuNPs stabilised by a thiol‐bearing hybrid organophosphoryl‐modified Wells–Dawson POM, K_6_[P_2_W_17_O_61_(PO_2_C_17_H_26_−SH)_2_] (**1**),^14^ and report a detailed comparison of the new Au@hybrid‐POM nanoparticle composite **NP‐1** with its electrostatically stabilised analogue **NP‐P_2_W_18_**, comprising AuNPs noncovalently capped with the conventional K_6_[P_2_W_18_O_62_] (**{P_2_W_18_}**) Wells–Dawson cluster (Scheme 1).^15^ Transmission electron microscopy (TEM), X‐ray photoelectron spectroscopy (XPS), and X‐ray absorption near‐edge structure (XANES), UV/Visible absorption, and FT‐IR analyses have been used to probe the structure and surface interactions of both nanoparticle composites, whilst spectroscopic and voltammetric analyses have been used to compare their photo‐ and electrochemical properties, respectively. Thus, this study represents the first detailed analysis of the vital role played by direct chemical attachment of the multifunctional POM capping agent to the nanoparticle surface.

**Scheme 1:**
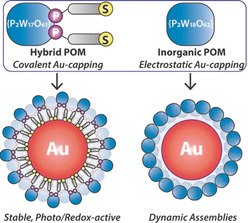
A route to stable redox‐ and photo‐active NP@POM composites.

The synthesis of the hybrid‐POM capping ligand, **1**, was carried out using a modified version of the synthesis previously described by our group.^14^ Briefly, 4‐((11‐(thio)undecyl)oxy)phenylphosphonic acid and the monolacunary precursor K_10_[P_2_W_17_O_61_] were combined by an acid‐mediated condensation reaction in acetonitrile (see the Supporting Information). The hybrid‐Au@POM composite **NP‐1** was then synthesised by mixing **1** with a 10 nm solution of citrate‐stabilised AuNPs (**Au@citrate**) in H_2_O and stirring of the mixture for 2 days at room temperature. The resulting composite nanoparticles had an Au‐to‐W ratio of 34:1, and the average number of POMs per AuNP was found to be approximately 400 (according to inductively coupled plasma optical emission spectroscopy; see the Supporting Information). **NP‐P_2_W_18_** was synthesised according to the same method by using **{P_2_W_18_}** in place of **1**.

TEM of both nanocomposites showed that **NP‐1** comprised monodisperse and well‐separated NPs with an average diameter (*d*_all_) of 11.0 nm (Figure 1). Owing to the excellent contrast of W in TEM imaging, a clear surface layer of POMs could be resolved as a ring around the surface of the AuNPs in **NP‐1**. This layer is estimated to have a thickness of 1.7 nm, which corresponds closely to the expected dimensions of a monolayer of hybrid‐POM clusters. No such effect was observed for **NP‐P_2_W_18_**, in which agglomerated groups of NPs (*d*=7.2 nm) with a similar shape to the Au@citrate nanoparticle precursors were observed (see Figure S22–24 in the Supporting Information). This result suggests that weaker or unstable coverage of the nanoparticle surface resulted when the electrostatic capping approach was used (in agreement with the aggregation observed in TEM imaging of these materials).

**Figure 1:**
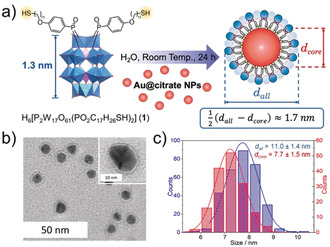
a) Synthesis of **NP‐1** ({WO_6_}: blue polyhedra, {PO_4_}: purple polyhedra, Au nanoparticles: red spheres, hybrid POM **1**: blue spheres, cations omitted for clarity). b) TEM image of **NP‐1** highlighting the surface‐bound monolayer of POMs (inset). c) Size distributions for **NP‐1** determined by TEM analysis.

FTIR spectroscopy of **NP‐1** showed similar features to those of the hybrid‐POM **1** owing to the excellent surface coverage of **1** on the AuNPs, whereas **NP‐P_2_W_18_** more closely resembled the citrate‐capped AuNPs (see Figures S11 and S12).^16^ The UV/Visible spectrum of **NP‐1** also exhibited features consistent with successful formation of the nanocomposite: a strong absorption centred at 335 nm corresponding to the ligand‐to‐metal charge transfer (LMCT) band of the hybrid POM and a surface‐plasmon resonance (SPR) peak at 531 nm (see Figure S15).^17^ The SPR peak is centred at a slightly longer wavelength than that of the citrate‐stabilised AuNP precursors (527 nm) owing to the different refractive indices of the capping agents, thus confirming the successful capping of the AuNPs with **1**. The SPR peak of **NP‐P_2_W_18_** was found to be broader than that of the citrate‐capped NPs and was red‐shifted by about 15 nm (see Figure S18), which is consistent with agglomeration of the NPs as revealed previously by TEM.

The stability of both composites was compared under a range of different conditions. The thermal stability of both materials was studied by UV/Visible spectroscopic analysis. Whereas the plasmon resonance band of **NP‐1** showed no change after heating at 80 °C for 24 h, that of **NP‐P_2_W_18_** disappeared (see Figures S32 and S33). Furthermore, **NP‐1** was stable upon storage for 1 month at −6 °C, and for at least 24 h in both 0.5 % H_2_O_2_ and buffer solutions (pH 5 and 7; measured at 37 °C). Conversely, **NP‐P_2_W_18_** degraded under all of these conditions (see Figures S34–S38). The excellent stability of **NP‐1** in a range of environments (including under biologically relevant conditions) suggests that **NP‐1** and similar covalently stabilised NP@POM materials could find use in a range of potentially demanding applications, such as sensing or catalysis.

Following basic characterisation and comparison of our systems, we probed the interactions between the capping POM groups and nanoparticle surfaces. XPS was performed as a means to investigate the Au–POM interaction in both systems (Figure 2 a). The Au 4f core XP spectrum of **NP‐P_2_W_18_** shows significantly lower binding energies (83.3 and 87.0 eV) when compared to **NP‐1** (84.0 and 87.7 eV), thus providing further evidence that the capping agents in **NP‐P_2_W_18_** have a strong electrostatic interaction with the Au surface. Similar binding energies were also recently found by Weinstock and co‐workers for analogous electrostatically decorated Au@{P_2_W_18_} NPs (83.5 and 87.2 eV).6d Typically, lower binding energies point towards decreased oxidation states or, more accurately in this case, a higher negative surface polarisation/charge density resulting from the capping of the AuNPs by the negatively charged POM in **NP‐P_2_W_18_**.6d, 18 It is therefore interesting that the binding energies found in **NP‐1** are higher and are an excellent match to the values expected for both thiol‐functionalised Au^0^ surfaces^19^ and bulk Au^0^ ((84.0±0.2) and (87.7±0.2) eV). This result suggests that the POM clusters are shielded from the Au surface by the ligand groups, which helps to minimise the electrostatic interaction between them. A similar effect has been reported by Cronin, Kadodwala, and co‐workers for POM monolayers on gold surfaces.^20^

**Figure 2:**
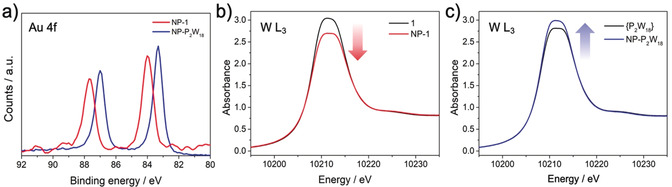
a) XP spectra of **NP‐1** and **NP‐P_2_W_18_** showing the Au 4f_7/5_ and 4f_7/2_ absorptions, respectively. b) XANES spectra of the hybrid POM **1** and the nanocomposite **NP‐1**, showing a decrease in the intensity of the W L_3_ “white‐line” absorption edge upon formation of the hybrid nanomaterial. c) XANES spectra of **{P_2_W_18_}** and **NP‐P_2_W_18_**, showing an increase in the intensity of the W L_3_ absorption edge upon interaction of the POMs with Au.

To further probe the surface interactions of the POM‐capped nanomaterials, XANES analysis was performed on both **NP‐1** and **NP‐P_2_W_18_** and compared to the corresponding spectra for the native POM cluster in each case (Figure 2 b,c). The broad “white‐line” W L_3_‐edge observed in all four spectra corresponds well to the expected peak shape for distorted {WO_6_} octahedra.^21^ More interestingly, the intensity of the white‐line signal can also provide an indication of the charge density associated with the W centres, for which a clear difference can be observed between the covalently bound species in **NP‐1** and the electrostatically bound clusters in **NP‐P_2_W_18_**. The white‐line intensity falls in the case of the former, suggesting an increase in the effective charge density of the W centres in the hybrid POM, whereas it rises in the case of the latter, suggesting a reduction in the charge density. This result supports the findings of the XPS analysis, suggesting that each POM interacts differently with the Au surface. In the case of the noncovalent composites, **{P_2_W_18_}** donates electron density in a manner similar to that previously discussed by Weinstock and co‐workers.6d As discussed above, however, the covalently bound POMs in **NP‐1** are effectively shielded from the gold surface and do not electronically interact with it. Instead, one possibility is that the tight packing of the POM monolayer around the NPs (which have a notably high POM loading) increases the coulombic interactions between neighbouring anions, thus significantly modifying the local environment of each cluster. This hypothesis agrees with previous observations we have made on micellar systems,^14^ and also with previous studies of POM monolayers formed on AuNPs, where even the extensive integration of countercations into the monolayer does not fully attenuate the high negative charge at the composite surface.6c This behaviour is likely to have profound implications for the photochemical and redox properties of the composite materials (see below).

The photochemical properties of the new nanocomposites were evaluated by irradiating a deaerated sample of **NP‐1** in *N*,*N*′‐dimethylformamide (DMF) using a solar simulator (200 W) fitted with a 395 nm cutoff filter. After visible‐light irradiation for 30 min, the absorption spectrum of **NP‐1** exhibited the typical intervalence charge transfer (IVCT) band expected for a monoreduced organophosphoryl hybrid Wells–Dawson ion at 820 nm,8a whereas the SPR band from the AuNP core at 531 nm remained intact (Figure 3). Interestingly, the sample could be readily and cleanly reoxidised by bubbling O_2_ into the solution, and several reduction–oxidation cycles could be performed without any evidence of decomposition (see Figure S30), thus indicating that these materials may be useful for photometric or photocatalytic applications.^22^ In contrast, the photoreduced state of **NP‐P_2_W_18_** could only be accessed using more aggressive UV irradiation (i.e. <395 nm), under which conditions **NP‐P_2_W_18_** decomposed (see Figure S31). Although organophosphonate hybrid‐POM clusters in particular are known to be photoactive in the near‐visible regime, their photoreduction can be difficult to reverse in O_2_ owing to their low‐lying LUMO levels and correspondingly more positive redox potentials.^23^ One possibility is that the tightly packed structure of **1** on the NP surface may destabilise the reduced state of the POM, as seen previously in micellar systems,^14^ thus facilitating its reoxidation by a relatively mild oxidant (O_2_).

**Figure 3:**
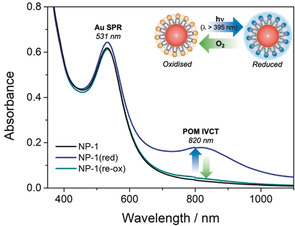
UV/Visible absorption spectra of **NP‐1** in DMF after photoreduction of capping POMs with visible light (blue) and after subsequent aerobic oxidation and recovery of the native state (green).

Owing to the highly reversible, rich multielectron redox chemistry conferred by the POM capping groups, we also studied the electrochemical properties of both composites. Cyclic voltammetry of 0.125 mm **NP‐1** in DMF showed two quasireversible redox processes, centred at −0.76 V (peak‐to‐peak separation, Δ*E*_p_=90 mV) and −1.15 V (Δ*E*_p_=140 mV) versus Fc/Fc^+^ (Figure 4). This cyclic voltammogram is similar to that of the discrete hybrid POM **1** (*E*_1/2_=−0.74 V (Δ*E*_p_=70 mV) and −1.12 V (Δ*E*_p_=100 mV); Figure 4). The larger Δ*E*_p_ values and negatively shifted redox potentials observed for **NP‐1** indicate that reduction/oxidation is more kinetically and thermodynamically hindered in this system. This result agrees with both the XANES analysis, which indicates that **1** is more electron‐rich when bound to the NP surface (Figure 2 b), and our previous discussion around the aerobic reoxidation of the photoreduced state. **NP‐1** was also shown to be stable to repeated potential cycling (see Figure S20), thus highlighting the strong attachment of the POM to the Au surface. In contrast, the behaviour of **NP‐P_2_W_18_** is identical to that of the **{P_2_W_18_}** capping groups (see Figure S19), indicating that the kinetics of electron transfer to and from the weakly bound POMs appear unaffected by their electrostatic association with the AuNP surface.

**Figure 4:**
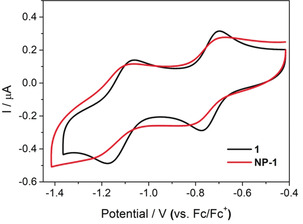
Cyclic voltammograms of 0.125 mm solutions of **1** and **NP‐1** in DMF at a scan rate of 100 mV s^−1^. Initial scan was to negative potentials. Potentials have been reported relative to that of the Fc/Fc^+^ redox couple.

The electrochemical properties of **1** and **NP‐1** were also compared by voltammetry using a Pt ultramicroelectrode (see the Supporting Information for details). POM **1** yielded a clear steady‐state voltammogram owing to efficient, convergent diffusion of the redox species to the electrode surface from the expanding diffusion field. In contrast, **NP‐1** yielded a transient (peak‐shaped) response due to depletion of the slowly diffusing nanocomposite within the diffusion field next to the electrode surface, consistent with the retention of stable POM–NP interactions in **NP‐1**.

In summary, we have successfully prepared highly stable AuNPs functionalised with covalently bound organic–inorganic hybrid POMs. Extensive characterization of the new nanomaterial **NP‐1** was performed using a range of spectroscopic and imaging techniques, which demonstrated unambiguously that hybrid POMs are directly attached to the AuNP surface through a thiolate‐type binding mode. The thermal, chemical, photochemical, and electrochemical stability of the new covalent nanocomposite was investigated through detailed comparison with the electrostatically functionalised analogue **NP‐P_2_W_18_**, which clearly demonstrated the crucial and highly effective role that covalent functionalisation plays in stabilising the assembly. This multifunctional nanomaterial was found to combine the optical properties of gold nanoparticles with the electro‐ and photochemical features of POMs. The rich, reversible electrochemistry and remarkable, efficient, and reversible photoactivation under visible light means that these materials may find new application in a range of catalytic and sensing technologies. Future studies will explore how more systematic approaches to the molecular design of the hybrid POM, in conjunction with the nanoparticle size, shape, and composition, can be used to both tailor and improve the properties of new composite materials.

## Conflict of interest

The authors declare no conflict of interest.

## Supporting information

As a service to our authors and readers, this journal provides supporting information supplied by the authors. Such materials are peer reviewed and may be re‐organized for online delivery, but are not copy‐edited or typeset. Technical support issues arising from supporting information (other than missing files) should be addressed to the authors.

Supplementary
