# Supplementary material for: Redox‐Active Hybrid Polyoxometalate‐Stabilised Gold Nanoparticles
Source: Angew Chem Int Ed Engl. 2020 Jun 29;59(34):14331–5. doi: 10.1002/anie.202005629 (PMC7497208; doi:10.1002/anie.202005629)
Supplement: Supplementary file 1 — Supplementary [file ANIE-59-14331-s001.pdf]

Angewandte Chemie
Eine Zeitschrift der Gesellschaft Deutscher Chemiker

Supporting Information

Redox-Active Hybrid Polyoxometalate-Stabilised Gold Nanoparticles

Carmen Martin, Katharina Kastner, Jamie M. Cameron, Elizabeth Hampson, Jesum Alves Fernandes, Emma K. Gibson, Darren A. Walsh, Victor Sans,* and Graham N. Newton**

anie_202005629_sm_miscellaneous_information.pdf

SUPPLEMENTARY INFORMATION

1. General Considerations S3
2. Synthetic Procedures S5
3. NMR Spectra S10
4. IR Spectra S14
5. UV-Vis Spectra S17
6. Electrochemistry S20
7. TEM Images S23
8. Calculation of Average Number of POMs per Nanoparticle S26
9. Stability Studies of **NP-1** and **NP-P_2W_{18}** S28

1. GENERAL CONSIDERATIONS

All oxygen and moisture sensitive operations were carried out under argon atmosphere using standard vacuum-line and Schlenk techniques. Solvents were purchased from Sigma-Aldrich as HPLC grade and dried by means of an Inert Pure solv MD purification system. All reagents were purchased from commercial suppliers (Aldrich and Acros) and used as received. $K_6[P_2W_{18}O_{62}]$ and $K_{10}[P_2W_{17}O_{61}]$ were prepared according to literature methods.[1] AuNPs stabilized by citrate (10 nm) was purchased from Sigmal Aldrich.

NMR spectra were recorded on a Bruker AV3400 400 MHz and Bruker DPX500 500 MHz nuclear magnetic resonance spectrometerand referenced to the residual NMR solvent signals. Signals are quoted as s (singlet), d (doublet), dd (double doublet), m (multiplet), b (broad).

UV-vis spectroscopy of all samples was performed on a Cary UV Vis NIR Spectometer.

ATR FT-IR measurements were performed on a Bruker Alpha Series FT-IR spectrometer equipped with an attenuated total reflectance (ATR) module. The ATR FT-IR spectra were recorded by collecting 16 scans of a compound in the ATR module.

Electrochemical measurements were performed on a CHI600e (CH Instruments) workstation. Full details of experimental conditions are provided below (pages S20 and S22).

Mass spectra of the organic compounds were recorded on a Bruker MicroTOF spectrometer ionised by electrospray ionisation (ESI). Additional mass analyses were performed on a gas chromatography mass spectrometry (GC-MS) ThermoTraceGold TG SQC equivalent to TG5MS (15m x 0.25mm x 25um) equipped with a Single Quadrupole Mass Spectrometer.

High Resolution Transmission Electron Microscopy (HRTEM) was performed using a JEOL 2100F operating at an accelerating voltage of 200 kV.

---

[1] R. Contant, W. G. Klemperer, O. Yaghi. Potassium Octadecatungstodiphosphates(V) and Related Lacunary Compounds. In Inorganic Syntheses; John Wiley & Sons, Inc., 2007, 104−111.

X-ray photoelectron spectroscopy (XPS) measurements were performed using a Kratos AXIS Ultra DLD instrument. The chamber pressure during the measurements was 5 × 10−9 Torr. Wide energy range survey scans were collected at pass energy of 80 eV in hybrid slot lens mode and a step size of 0.5 eV. High-resolution data on the C 1s, O 1s, S 2p, P 2p, Au 4f and W 4f photoelectron peaks was collected at pass energy 20 eV over energy ranges suitable for each peak, and collection times of 5 min, step sizes of 0.1 eV. The charge neutraliser filament was used to prevent the sample charging over the irradiated area. The X-ray source was a monochromated Al K_α emission, run at 10 mA and 12 kV (120 W). The energy range for each 'pass energy' (resolution) was calibrated using the Kratos Cu $2p_{3/2}$, Ag $3d_{5/2}$ and Au $4f_{7/2}$ three-point calibration method. The transmission function was calibrated using a clean gold sample method for all lens modes and the Kratos transmission generator software within Vision II. The data were processed with CASAXPS (Version 2.3.17). The high resolution data was charge corrected to the reference carbon adventitious signal at 284.8 eV.

XANES spectroscopy measurements were performed on the I20-scanning beamline at the Diamond Light Source (Harwell Campus, UK). I20 is equipped with a Si(111) four bounce monochromator for selecting the incident X-ray energy, a multi-element Ge (fluorescence) detector was used to record the X-ray absorption spectra. The measurements were performed in air at room temperature at the W L_3 edge. Multiple scans were taken to improve signal-to-noise ratio. All data were analysed using the Athena software package.[2]

Irradiation experiments were performed with a solar simulator ORIEL instrument, model 66902, with a power of 200 W.

Inductively Coupled Plasma Optical Emission Spectrometry (ICP-OES). The gold and tungsten content determination was performed through analysis of the samples by ICP-OES on a Perkin Elmer Optima 2000 DV ICP-OES. The samples were prepared by digestion of 3 mg of dry gold nanoparticles in *aqua regia* (1 mL, HCl/HNO_3, 1:3 v/v) overnight. The digestion mixture was diluted with a solution of HCl 5 %. Calibration curves were prepared using standard solutions of concentrations between 70 to 230 ppm for gold and 5 to 40 ppm for tungsten prepared by dilution of the standard solution. The gold and tungsten Standard for ICP TraceCERT® (999 mg/L Au in hydrochloric acid and 1'000 mg/L W in nitric acid and hydrofluoric acid) were supplied by Sigma Aldrich.

---

[2] B. Ravel and M. Newville, *J. Synchro. Rad.*, **2005**, *12*, 537–541.

2. SYNTHETIC PROCEDURES

The synthesis of ligand (**D**) was carried out according to a modified literature procedure.[3]

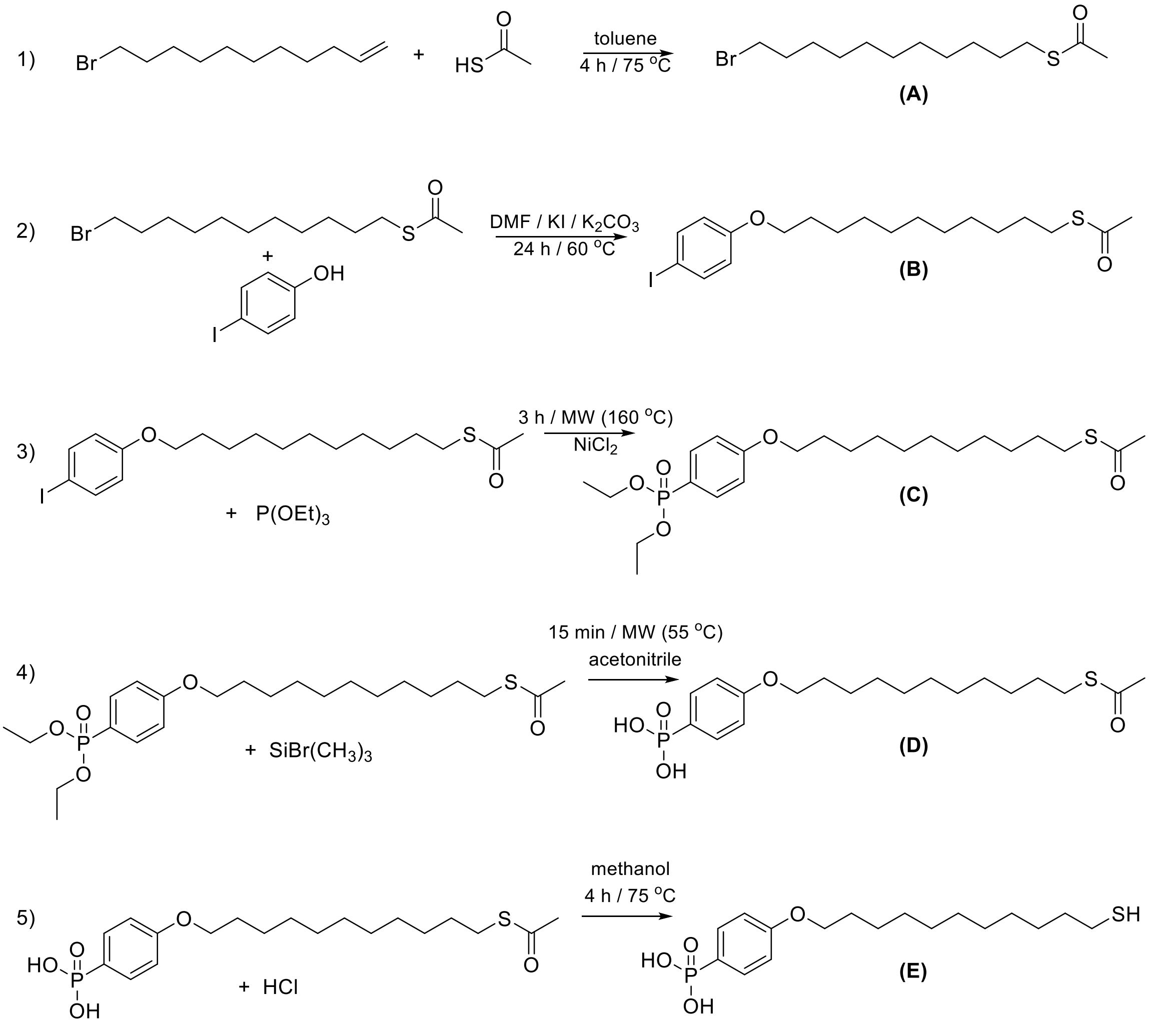

Synthesis of (11-bromoundecyl)ethanethiolate (A). 2,2'-azobis(2-methylproprionitrile) (AIBN) (1.2 g, 8.0 mmol, 0.2 eq) was dissolved in 100 mL of dry toluene under inert atmosphere and 11-bromo-1-undecene (8.8 mL, 40.0 mmol, 1.0 eq) was added into the solution. After careful addition of thioacetic acid (8.6 mL, 120.0 mmol, 3.0 eq) the solution was stirred at 75 °C for 4h. Then, the reaction mixture was cooled down to room temperature and washed with saturated $NaHCO_3$ solution (3 x 100 mL) and deionised water (100 mL). The organic layer was dried over $MgSO_4$ and toluene was removed in *vacuo* to give a yellow residue that was purified via silica column chromatography (petroleum ether/ethyl acetate,

[3] G. N. Newton, V. Sans, *et al. J. Mater. Chem. A,* **2017**, *5*, 11577-11581.

v/v = 10/1). Yield: 6.9 g (30.0 mmol, 77 %). ^{1}H NMR (400 MHz, $CDCl_3$): δ = 3.39 (t, $^3J_{HH}$ = 6.9 Hz, 2H, BrCH_2), 2.85 (t, 2H, $^3J_{HH}$ = 7.4 Hz, SCH_2), 2.31 (s, 3H, C(O)CH_3), 1.88-1.79 (m, 2H, CH_2), 1.59-1.50 (m, 2H, CH_2), 1.46-1.23 (m, 14H, CH_2). ^{13}C NMR (101 MHz, $CDCl_3$): δ = 196.0 (1C, *C*=O), 34.0 (1C, Br*C*H2), 32.8 0 (1C, *C*H2), 30.6 (1C, *C*H3), 29.5 (1C, C(O)S*C*H2), 29.4 (3C, *C*H2), 29.1 (1C, *C*H2), 29.0 (1C, *C*H2), 28.8 (1C, *C*H2), 28.7 (1C, *C*H2), 28.2 (1C, *C*H2). MS 308.98 (M+1) (calcd. 309.09), MS 307.89 (M) (calcd. 308.08), 266.01 (M-$COCH_3$+1)(calcd. 266.07). Characteristic IR bands (cm^{-1}): 2924 (C-H), 2853 (C-H), 1690 (C=O), 624 (S-C).

Synthesis of (11-(4-iodophenoxy)undecyl)ethanethioate (B). A (6.7 g, 30.5 mmol, 1.0 eq) and 4-Iodophenol (6.8 g, 30.5 mmol, 1.0 eq), potassium iodide (750.0 mg, 4.6 mmol, 15 mol %) and potassium carbonate (8.4 g, 60.9 mmol, 2 eq) were dissolved in 75 mL of dry *N,N'*-dimethyl formamide under an inert atmosphere. The reaction mixture was heated at 60 °C for 24 h. Upon completion of the reaction the solvent was removed in *vacuo*. The crude was dissolved in diethyl ether (50 ml) and washed with 2 x 30 mL of NaOH 2 M and 1 x 30 mL of brine. The organic phase was dried over $MgSO_4$ and concentrated at the rotary evaporator to give a yellow residue that was purified via silica column chromatography (petroleum ether/ethyl acetate, v/v = 10/1). Yield: 6.5 g (14.3 mmol, 47 %). ^{1}H NMR (500 MHz, $CDCl_3$): δ = 7.56-7.52 (m, 2H, CH_{Ar}), 6.70-6.65 (m, 2H, CH_{Ar}), 3.91 (t, $^3J_{HH}$ = 6.3 Hz, 2H, OCH_2), 2.87 (t, 2H, $^3J_{HH}$ = 7.3 Hz, SCH_2), 2.33 (s, 3H, C(O)CH_3), 1.80-1.73 (m, 2H, CH_2), 1.61-1.54 (m, 2H, CH_2), 1.48 – 1.26 (m, 14H, CH_2). ^{13}C NMR (126 MHz, $CDCl_3$): δ = 195.9 (1C, *C*=O), 159.0 (1C, C_{Ar}), 138.1 (2C, CH_{Ar}), 116.9 (2C, CH_{Ar}), 82.4 (1C, C_{Ar}), 68.1 (1C, O*C*H2), 32.2 (1C, *C*H2), 30.7 (1C, *C*H3), 29.5 (1C, C(O)*C*H2), 29.5 (2C, *C*H2), 29.4 (1C, *C*H2), 29.2 (1C, *C*H2), 29.1 (1C, *C*H2), 28.8 (1C, *C*H2), 26.0 (1C, *C*H2). ESI-MS (+ ve): m/z = 471.0822 (M+ Na) (calculated: m/z = 471.0831). Characteristic IR bands (cm^{-1}): 2921 (C-H), 2851 (C-H), 1689 (C=O), 1586 (C_{Ar}=C_{Ar}), 1572 (C_{Ar}=C_{Ar}), 1486 (C_{Ar}=C_{Ar}), 1468 (C_{Ar}=C_{Ar}), 1241 (C-O-C), 625 (S-C), 504 (C-I).

Synthesis of (11-(4-(diethoxyphosphoryl)phenoxy)undecyl)ethanethioate (C). B (6.4 g, 14.3mmol, 1 eq), triethylphosphite (3.7 mL, 21.4 mmol, 1.5 eq) and anhydrous $NiCl_2$ (185.0 mg, 1.4 mmol, 10 mol %) were placed in a microwave tube with a stirring bar under an argon atmosphere. The mixture was heated under microwave conditions at 160 °C for 3h. The reaction mixture was poured into diethyl ether and the precipitate was filtered off. The solvent was removed in *vacuo* to give a yellow residue that was purified via silica column chromatography (chloroform/ethyl acetate v/v = 3/1). Yield: 5.2 g (11.4 mmol, 80 %). ^{1}H NMR (400 MHz, $CDCl_3$): δ = 7.75-7.67 (m, 2H, CH_{Ar}), 6.69-6.91 (m, 2H, CH_{Ar}), 4.14-4.00 (m, 4H, PCH_2), 3.98 (t, $^3J_{HH}$ = 6.7 Hz, 2H, OCH_2), 2.85 (t, 2H, $^3J_{HH}$ = 7.6 Hz, SCH_2), 2.31 (s, 3H, C(O)CH_3), 1.82-1.73 (m, 2H, CH_2), 1.59-1.50 (m, 2H, CH_2), 1.48-1.38 (m, 2H, CH_2),

1.37-1.22 (m, 12H, CH_2), 1.30 (t, 6H, $^3J_{HH}$ = 7.1 Hz, PCH_2CH_3). ^{13}C NMR (126 MHz, $CDCl_3$): δ = 195.9 (1C, C=O), 152.4 (d, $^4J_{CP}$ = 2.9 Hz, 1C, C_{Ar}), 138.7 (d, $^3J_{CP}$ = 11.0 Hz, 2C, CH_{Ar}), 119.1 (d, $^1J_{CP}$ = 119.1 Hz, 1C, C_{Ar}), 114.4 (d, $^2J_{CP}$ = 16.2 Hz, 2C, CH_{Ar}), 68.0 (1C, OCH_2), 61.8 (d, $^2J_{CP}$ = 5.4 Hz, 4C, P-OCH_2), 30.6 (1C, SC(O)CH_3), 29.5 (2C, CH_2), 29.4 (CH_2), 29.3 (1C, CH_2), 29.1 (1C, CH_2), 29.0 (1C, CH_2), 28.7 (1C, CH_2), 25.9 (1C, CH_2), 16.3 (d, $^2J_{CP}$ = 6.2 Hz, 1C, P-OCH_2CH_3). ^{31}P NMR (162 MHz, $CDCl_3$): δ = 19.8. ESI-MS (+ ve): m/z = 459.2323 (M+ H^+)(calculated: m/z = 459.2334). Characteristic IR bands (cm^{-1}): 2979 (C-H), 2925 (C-H), 2854 (C-H), 1691 (C=O), 1599 (C_{Ar}=C_{Ar}), 1504 (C_{Ar}=C_{Ar}), 1491 (C_{Ar}=C_{Ar}), 1244 (P=O), 1131 (P-O-C), 1021 (P-O-C), 954 (P-O-C), 832 (P-O), 792 (P-O), 626 (S-C).

Synthesis of (4-((11-(acetylthio)undecyl)oxy)phenyl)phosphonic acid (D). C (5.2 g, 11.4 mmol, 1 eq) was placed in a microwave tube with a stirring bar and dissolved in 20 mL of dry acetonitrile under an argon atmosphere. After addition of bromotrimethylsilane (6.0 mL, 7.0 g, 45.8 mmol, 4.0 eq) the reaction mixture was heated under microwave conditions at 55 °C for 15 min. Upon completion of the reaction the solvent was removed *in vacuo* yielding a pale yellow oil. A solvent mixture of 30 ml of MeOH/DCM (v:v = 1:1) was added and the solvent was again removed in *vacuo*. The resulting solid was recrystallized from a mixture of acetonitrile and diethyl ether (v/v = 1/2) to give a white powder. Yield: 2.2 g (7.0 mmol, 61 %). ^{1}H NMR (400 MHz, DMSO-d_6): δ = 7.61-7.54 (m, 2H, CH_{Ar}), 6.69-6.95 (m, 2H, CH_{Ar}), 3.99 (t, $^3J_{HH}$ = 6.6 Hz, 2H, OCH_2), 2.81 (t, 2H, $^3J_{HH}$ = 7.2 Hz, SCH_2), 2.31 (s, 3H, C(O)CH_3), 1.75-1.66 (m, 2H, CH_2), 1.53-1.44 (m, 2H, CH_2), 1.44-1.35 (m, 2H, CH_2), 1.35-1.22 (m, 12H, CH_2). ^{13}C NMR (101 MHz,DMSO-d_6):δ= 195.8 (1C, C=O), 161.1 (d, $^4J_{CP}$ = 2.8 Hz,1C, C_{Ar}), 132.8 (d, $^3J_{CP}$ = 11.3 Hz,2C, CH_{Ar}), 125.8 (d, $^1J_{CP}$ = 187.1 Hz 1C, C_{Ar}), 114.4 (d, $^2J_{CP}$ = 15.5 Hz, 2C, CH_{Ar}), 68.0 (1C, OCH_2),31.0 (1C, C(O)CH_3), 29.6 (1C, CH_2), 29.4 (1C, C(O)CH_2), 29.4 (2C, CH_2), 29.3 (1C, CH_2), 29.2 (1C, CH_2), 29.0 (1C, CH_2), 29.0 (1C, CH_2), 28.8 (1C, CH_2), 28.6 (1C, CH_2), 26.0 (1C, CH_2). ^{31}P NMR (162 MHz, DMSO-d_6): δ = 13.6. ESI-MS (- ve): m/z = 401.1557 (M – H) (calculated: m/z = 401.1152). Characteristic IR bands (cm^{-1}): 2917 (C-H), 2850 (C-H), 2630 (O-H), 1686 (C=O), 1600 (C_{Ar}=C_{Ar}), 1571 (C_{Ar}=C_{Ar}), 1508 (C_{Ar}=C_{Ar}), 1472 (C_{Ar}=C_{Ar}), 1110 (P-O), 1000 (P-O), 994 (P-O), 636 (S-C).

Synthesis of (4-((11-(thio)undecyl)oxy)phenyl)phosphonic acid (E). D (0.5 g, 2.5mmol, 1 eq) was placed in a round bottom flask with a stirring bar and dissolved in 25 mL of methanol. After addition of 2.1 mL of HCl (37 wt. %), the reaction mixture was heated at 75 °C for 4 h. Upon completion of the reaction the solvent was removed *in vacuo* yielding a white solid. The solid was then washed with water and finally, the product was dissolved in acetone (25 mL), dried over $MgSO_4$ and concentrated using a rotary evaporator to give a

white powder. Yield: 280 mg (0.78mmol, 63 %).^{1}H NMR (500 MHz, DMSO-d_6): δ = 7.62-7.54 (m, 2H, CH_{Ar}), 6.69-6.93 (m, 2H, CH_{Ar}), 3.99 (t, $^{3}J_{HH}$ = 6.6 Hz, 2H, OCH_2), 2.46 (dt, 2H, $^{3}J_{HH}$ = 7.0 Hz, $^{3}J_{HH}$ = 6.5 Hz,CH_2SH), 2.21 (t, 1H, $^{3}J_{HH}$ = 6.5 Hz, SH), 1.74-1.67 (m, 2H, CH_2), 1.56-1.49 (m, 2H, CH_2), 1.44-1.36 (m, 2H, CH_2), 1.36-1.22 (m, 12H, CH_2). ^{13}C NMR (126 MHz, DMSO-d_6): δ = 161.1 (d, $^{4}J_{CP}$ = 3.6 Hz, 1C, C_{Ar}), 132.8 (d, $^{3}J_{CP}$ = 11.3 Hz, 2C, CH_{Ar}), 126.0 (d, $^{1}J_{CP}$ = 188.2 Hz 1C, C_{Ar}), 114.4 (d, $^{2}J_{CP}$ = 15.2 Hz, 2C, CH_{Ar}), 68.0 (1C, OCH$_2$), 33.9 (1C, CH$_2$), 29.4 (3C, CH$_2$), 29.2 (1C, CH$_2$), 29.0 (2C, CH$_2$), 28.2 (1C, CH$_2$), 25.9 (1C, CH$_2$), 24.2 (1C, CH$_2$SH). ^{31}P NMR (202 MHz, DMSO-d_6): δ = 13.4. ESI-MS (- ve): m/z = 359.1460 (M – H) (calculated: m/z = 359.1446). Characteristic IR bands (cm^{-1}): 2918 (C-H), 2849 (C-H), 2630 (O-H), 1599 (C_{Ar}=C_{Ar}), 1571 (C_{Ar}=C_{Ar}), 1506 (C_{Ar}=C_{Ar}), 1465 (C_{Ar}=C_{Ar}), 1011 (P-O), 943 (P-O).

Synthesis of $K_6[P_2W_{17}O_{61}(PO_2C_{17}H_{26}SH)_2]$ (1). The synthesis of the hybrid-POM **1** was carried out according to a modified literature procedure.[2] **E** (175 mg, 0.5 mmol, 2.0 eq) and $K_{10}[P_2W_{17}O_{61}]$ (1.1 g, 2.4·10^{-1} mmol, 1.0 eq) were suspended in 35 mL of acetonitrile and 1 mL of deionised water. After addition of 405 µL of HCl 37 wt-% (4.9 mmol, 20.0 eq) the reaction mixture was stirred at 90 °C for 24 h in the absence of the light. Then, the solution was cooled down to room temperature and the solvent was concentrated at the rotary evaporator. The resulting solid was redissolved in acetone to get an orange solution containing a white solid in suspension. The solid was separated from the supernatant by centrifugation. Next the solvent was removed at the rotary evaporator. Finally, the orange solid obtained was washed with chloroform and diethyl ether to remove unreacted ligand and dried under vacuum. Yield: 75 % (980 g, 1.8·10^{-1} mmol). ^{1}H NMR (500 MHz, CD_3CN): δ = 8.02 (dd, $^{3}J_{HH}$ = 8.7 Hz, $^{3}J_{PH}$ = 13.5 Hz, 2H, CH_{Ar}), 7.02 (dd, $^{3}J_{HH}$ = 8.6 Hz, $^{4}J_{PH}$ = 2.8 Hz, 2H, CH_{Ar}), 4.05 (t, $^{3}J_{HH}$ = 5.9 Hz, 2H, OCH_2), 2.70 (t, 2H, $^{3}J_{HH}$ = 7.6 Hz, CH_2SH), 1.81-1.75 (m, 2H, CH_2), 1.69-1.61 (m, 2H, CH_2), 1.50-1.42 (m, 2H, CH_2), 1.41-1.28 (m, 12H, CH_2). ^{13}C NMR (126 MHz,CD_3CN): δ = 162.4 (d, $^{4}J_{CP}$ = 2.7 Hz, 1C, C_{Ar}), 134.6 (d, $^{3}J_{CP}$ = 12.4 Hz, 2C, CH_{Ar}), 123.1 (d, $^{1}J_{CP}$ = 204.4 Hz 1C, C_{Ar}), 114.7 (d, $^{2}J_{CP}$ = 16.6 Hz, 2C, CH_{Ar}), 68.9 (1C, OCH$_2$), 30.3 (1C, CH$_2$), 30.2 (2C, CH$_2$), 30.1 (1C, CH$_2$), 29.9 (2C, CH$_2$), 29.8 (1C, CH$_2$), 29.1 (1C, CH$_2$), 26.7 (2C, CH$_2$). ^{31}P NMR (202 MHz, CD_3CN): δ= 16.9, -11.1, -12.7. Elemental analysis for $C_{34}H_{54}K_6O_{65}P_4S_2W_{17}$·5$CH_3CN$·4$H_2O$ in wt% (calcd.): C: 9.97 (9.92), H: 1.59 (1.46), N: 1.33 (1.31). Characteristic IR bands (cm^{-1}): 3563 (O-H), 2918 (C-H), 2850 (C-H), 1597 (P-O), 1567 (C_{Ar}=C_{Ar}), 1504 (C_{Ar}=C_{Ar}), 1465 (C_{Ar}=C_{Ar}), 1137 (P-O), 1088 (P-O), 950 (W-O), 903 (W-O), 717 (W-O). λ_{max} (DMSO): 300 nm. 300 nm and 820 nm when **1** is reduced.

Synthesis of Au@POM nanocomposites NP-1 and NP-P_2W_{18} by ligand exchange. A standard procedure to prepare both nanocomposites was as follows: 0.02 mmol of polyoxometalate (around 100 mg of **1** for **NP-1** or **P_2W_{18}** for **NP-P_2W_{18}**) were placed in a flask and dissolved in 5 mL of milli-Q water. Then, 25 mL of citrate stabilized AuNPs (from Sigma Aldrich, 10 nm) were added. The mixture was stirred for 2 days at 25 °C in the absence of light. Afterwards, the red solutions containing a solid in suspension were centrifuged at 7000 rpm for 5 min to separate the waste solid from the solution containing AuNPs. Next, the solvent was concentrated using a rotary evaporator to give a deep red solution (around 1-2 mL volume). 3 mL of methanol was added to precipitate the AuNPs which were then separated from the supernatant by centrifugation. AuNPs were washed with a mixture of methanol:acetonitrile (1:1, 4 mL in total). This process was repeated three more times before drying under vacuum. Although **NP-1** and **NP-P_2W_{18}** are stable at ambient temperature, both samples were stored in the dark at 6 °C.

NP-1. Black-red powder: 5.0 mg. Characteristic IR bands (cm^{-1}): 3482 (O-H), 3124 (O-H), 2928 (C-H), 2846 (C-H), 1625 (C=O), 1601 (P-O), 1568 (C_{Ar}=C_{Ar}), 1506 (C_{Ar}=C_{Ar}), 1138 (P-O), 1086 (P-O), 955 (W-O), 911 (W-O), 730 (W-O). λ_{max}(DMSO): 335 nm and 531nm.

NP-P_2W_{18}. Black-purple powder: 3.0 mg. Characteristic IR bands (cm^{-1}): 3150 (O-H), 1643 (C=O). λ_{max} (DMSO): 275 nm and 543 nm.

3. NMR SPECTRA

3.1 NMR SPECTRA OF LIGAND (E)

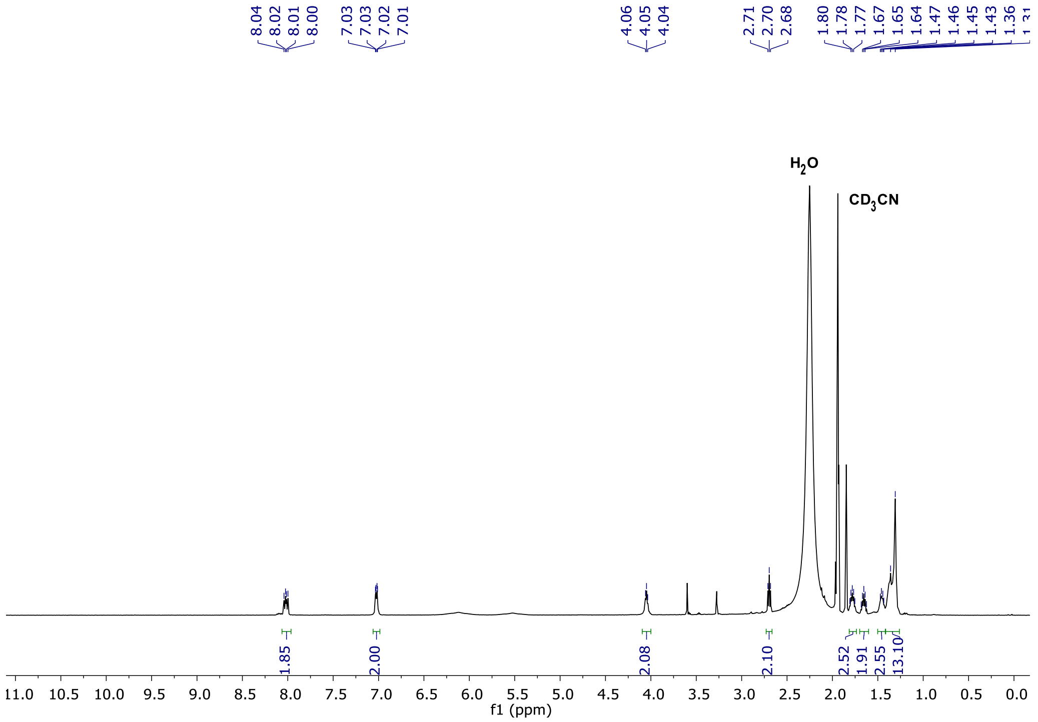

Figure S1. ^{1}H NMR spectrum of **(4-((11-(thio)undecyl)oxy)phenyl)phosphonic acid (E)** in DMSO-*d*6 (500 MHz, RT).

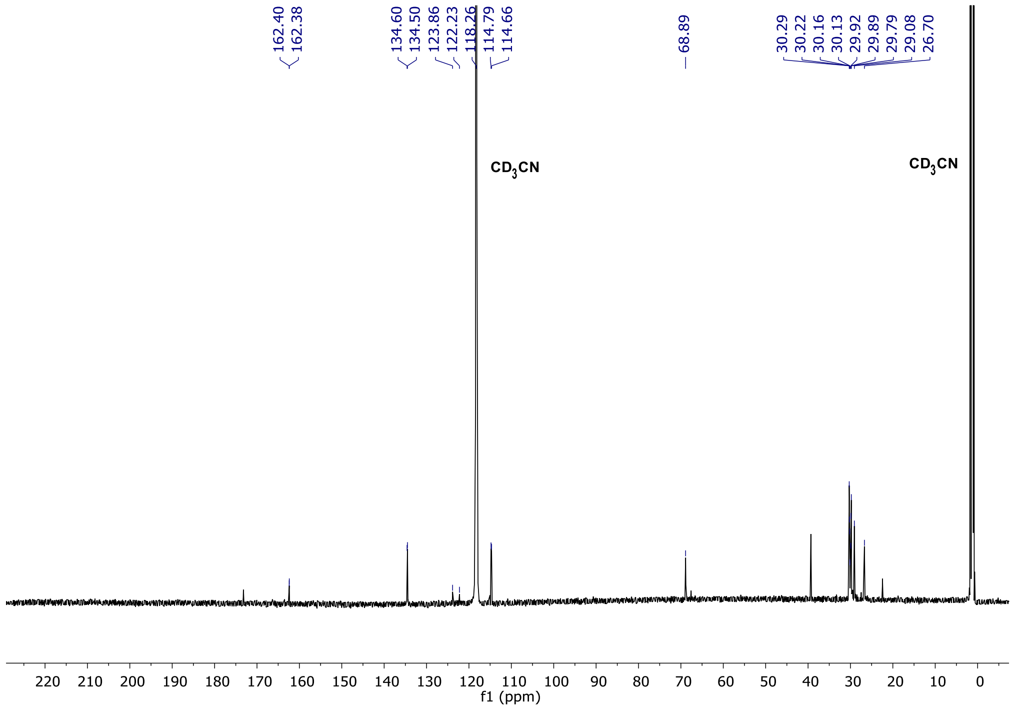

Figure S2. ^{13}C NMR spectrum of **(4-((11-(thio)undecyl)oxy)phenyl)phosphonic acid (E)** in DMSO-*d*6 (126 MHz, RT).

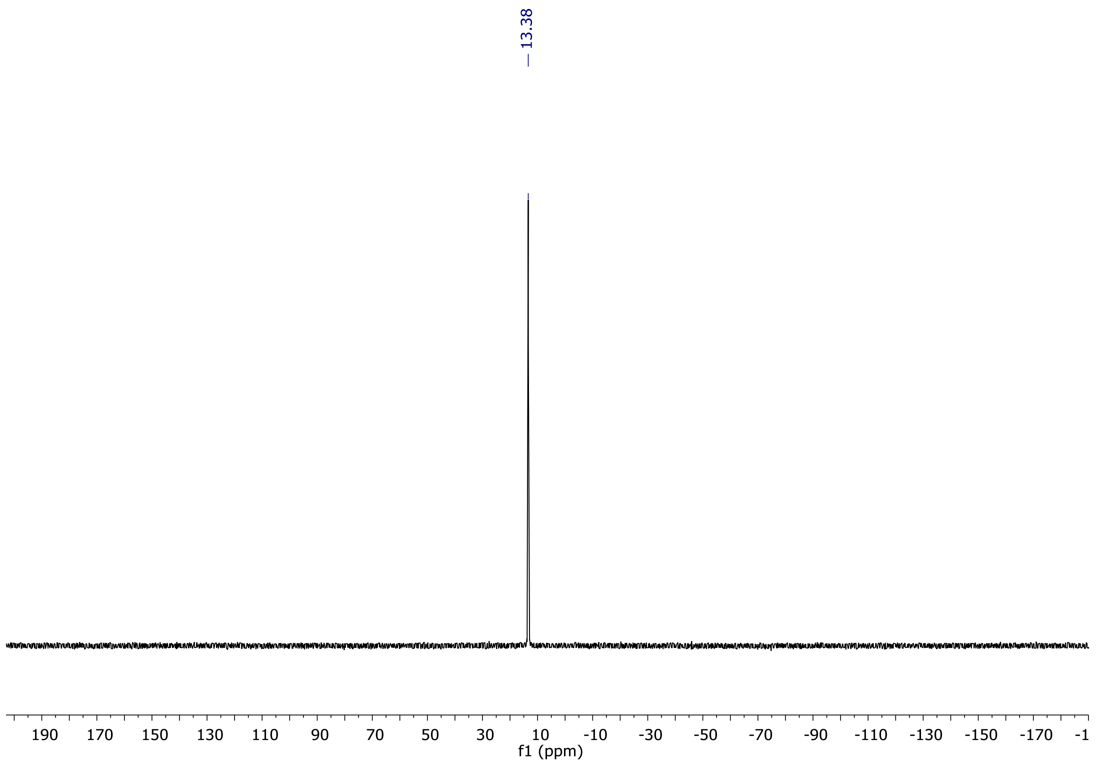

Figure S3. ^{31}P NMR spectrum of **(4-((11-(thio)undecyl)oxy)phenyl)phosphonic acid (E)** in DMSO-d_6 (202 MHz, RT).

3.2 NMR SPECTRA OF HYBRID-POM (1)

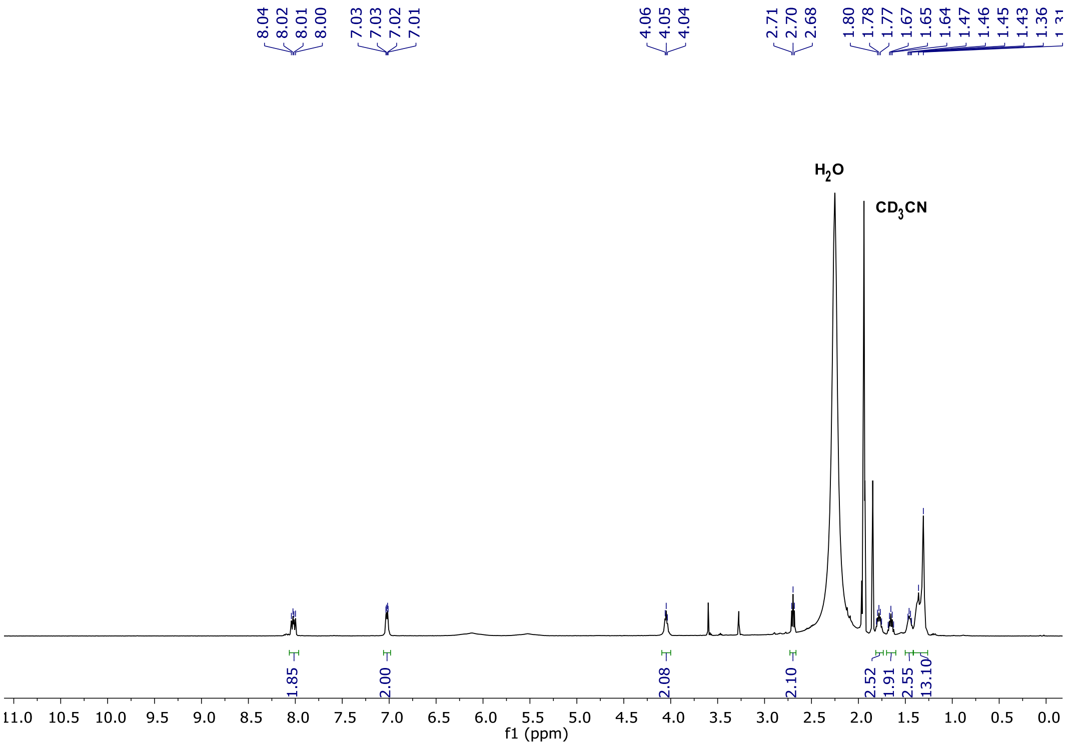

Figure S4. ^{1}H NMR spectrum of **1** in CD_3CN (500 MHz, RT).

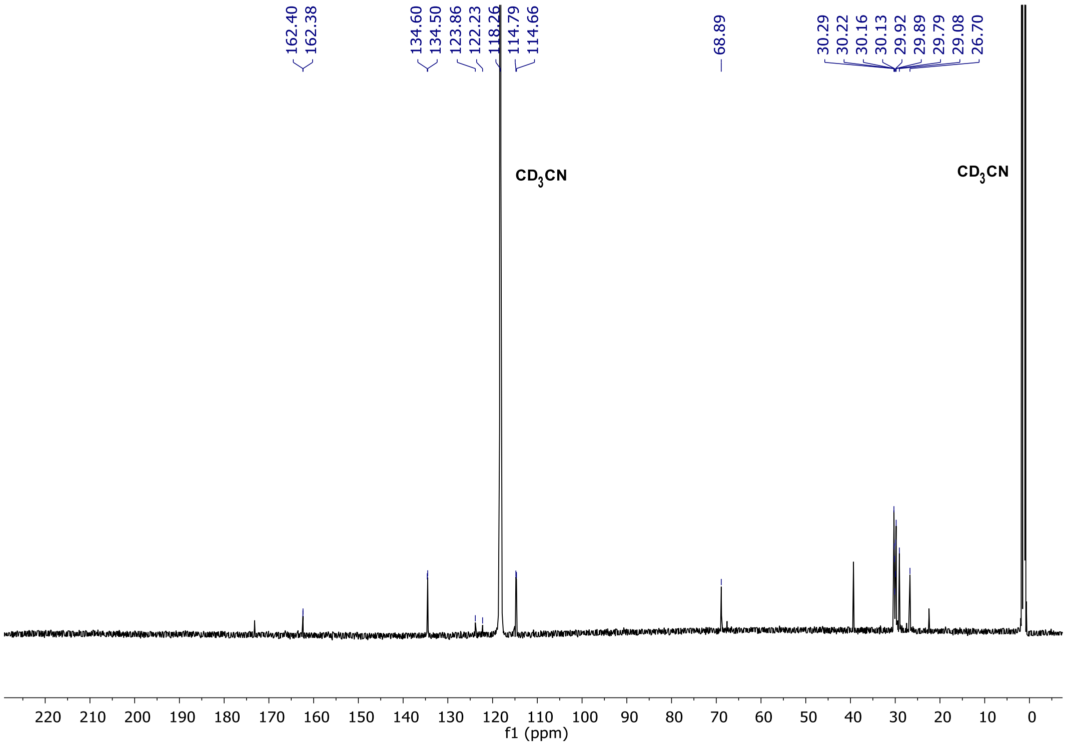

Figure S5. ^{13}C NMR spectrum of **1** in CD_3CN (126 MHz, RT).

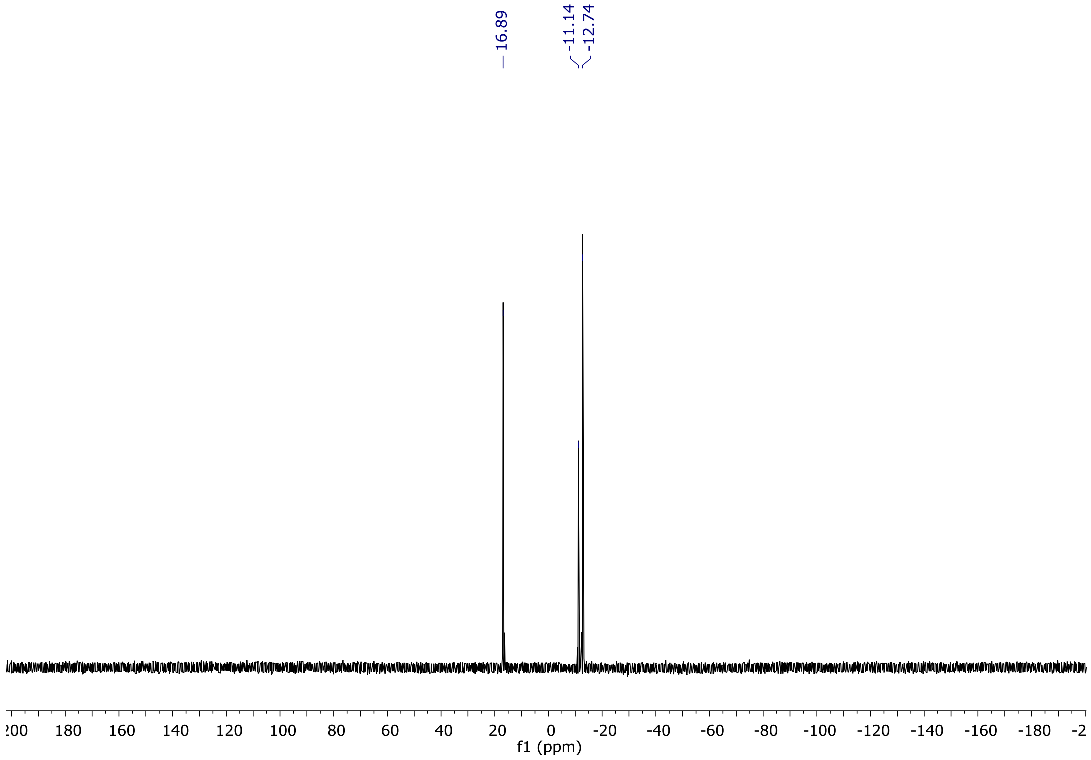

Figure S6. ^{31}P NMR spectrum of **1** in CD_3CN (202 MHz, RT)

4. IR SPECTRA

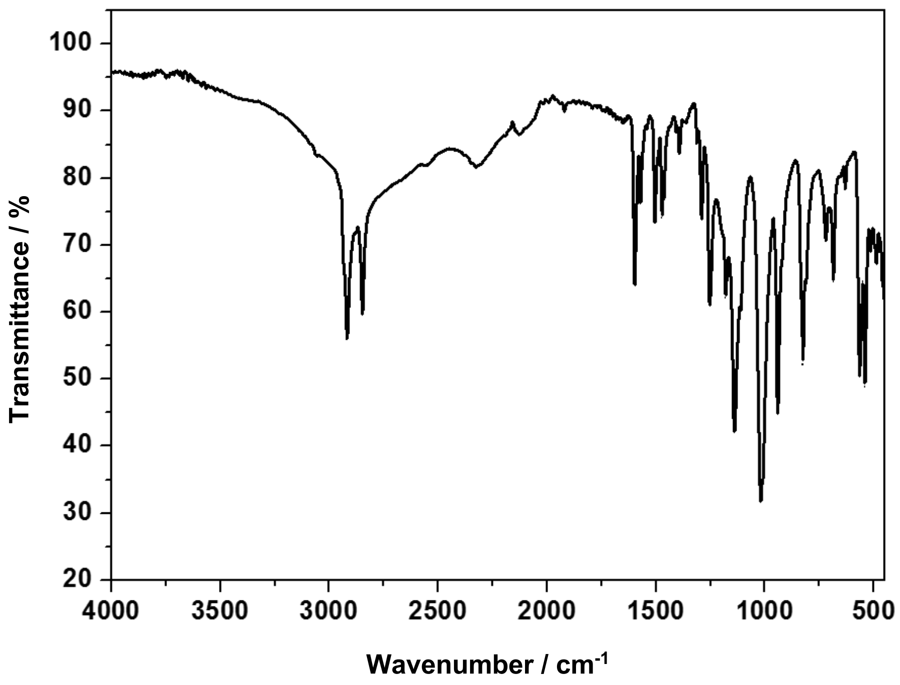

Figure S7. IR spectrum of **(4-((11-(thio)undecyl)oxy)phenyl)phosphonic acid (E)**.

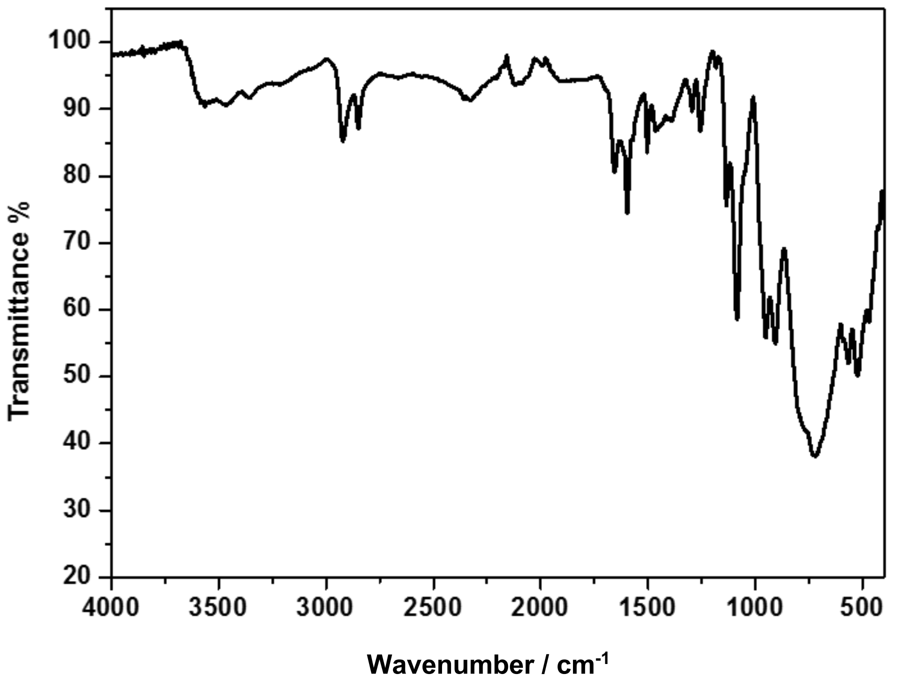

Figure S8. IR spectrum of **1**.

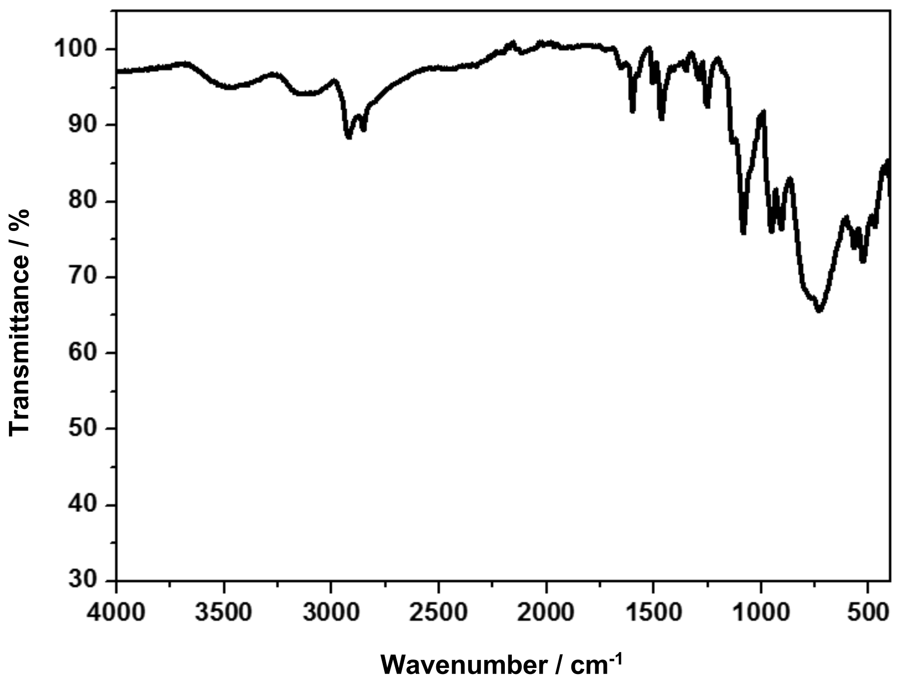

Figure S9. IR spectrum of **NP-1**.

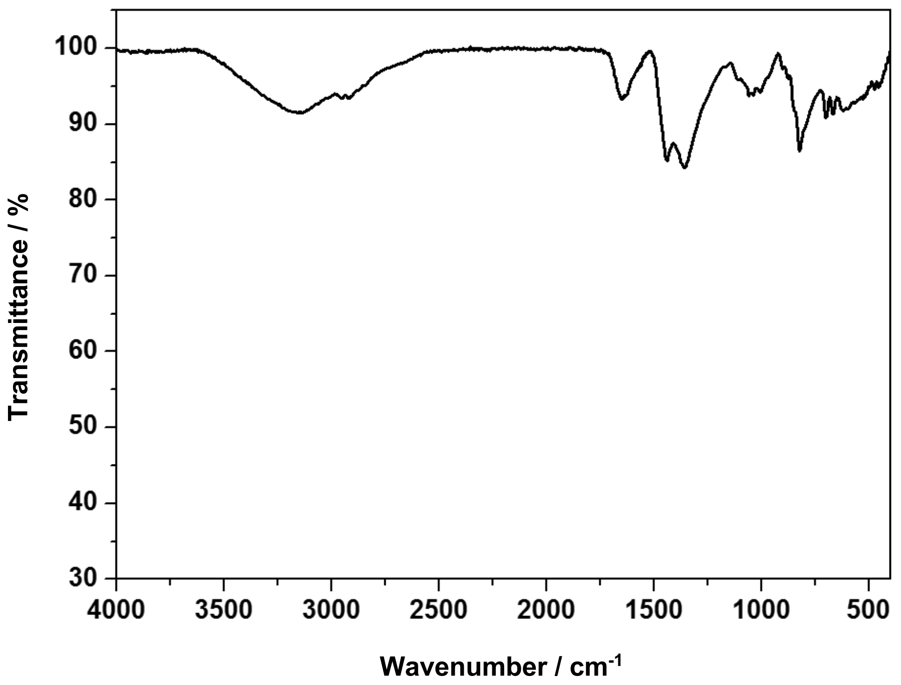

Figure S10. IR spectrum of **NP-P_2W_{18}**.

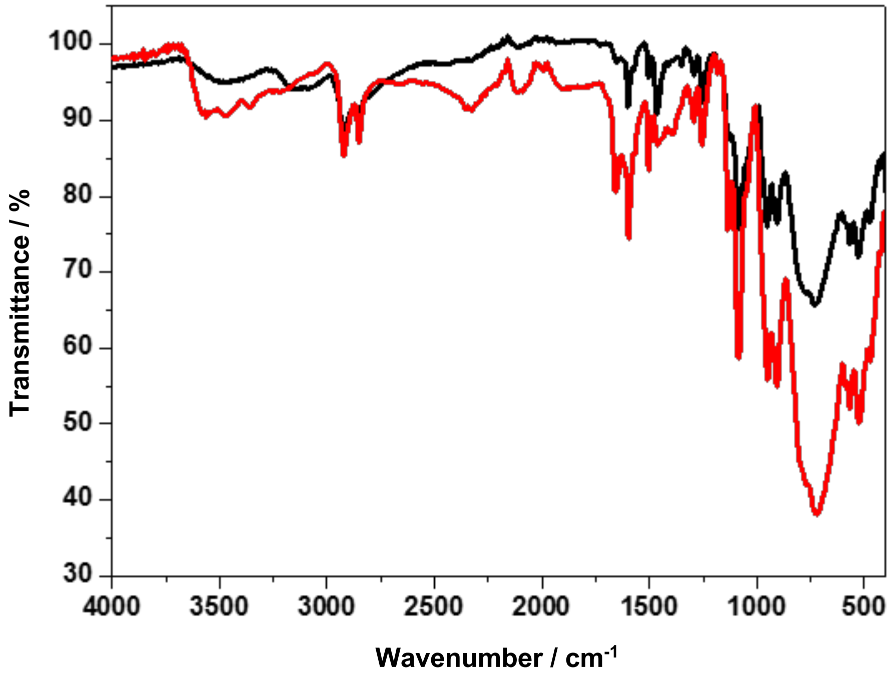

Figure S11. Comparative IR spectra for **1** (red trace) and **NP-1** (black trace).

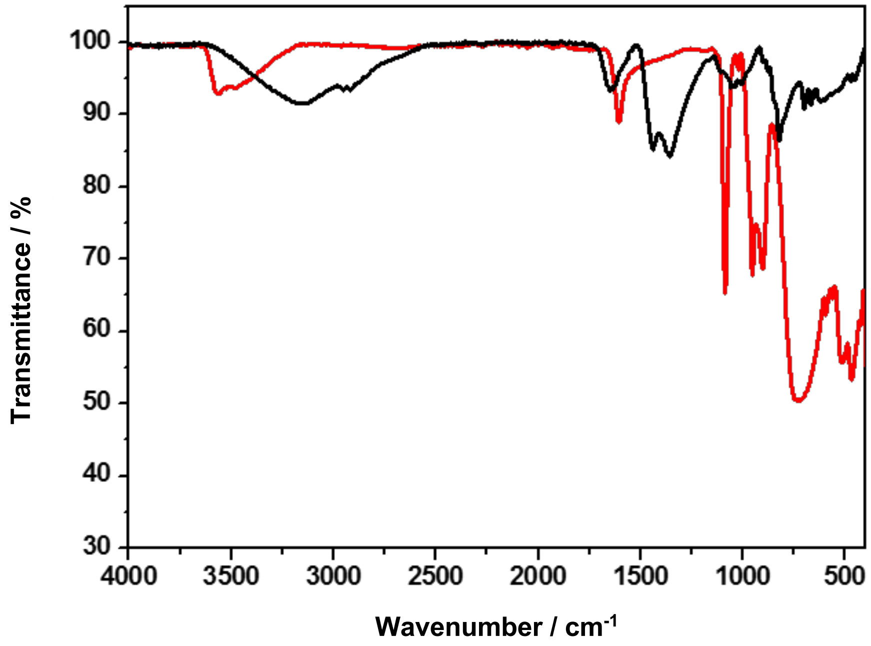

Figure S12. Comparative IR spectra for **{P_2W_{18}}** (red trace) and **NP-P_2W_{18}** (black trace). Note that the IR for **NP-P_2W_{18}** is quite similar to that reported for AuNPs@citrate.[4]

[4] Y.-C. Chen, I.-L. Lee, Y.-M. Sung, S.-P. Wu, *et al. Sensors and Actuators B* **2013**, *188*, 354-359.

5. UV-VIS SPECTRA

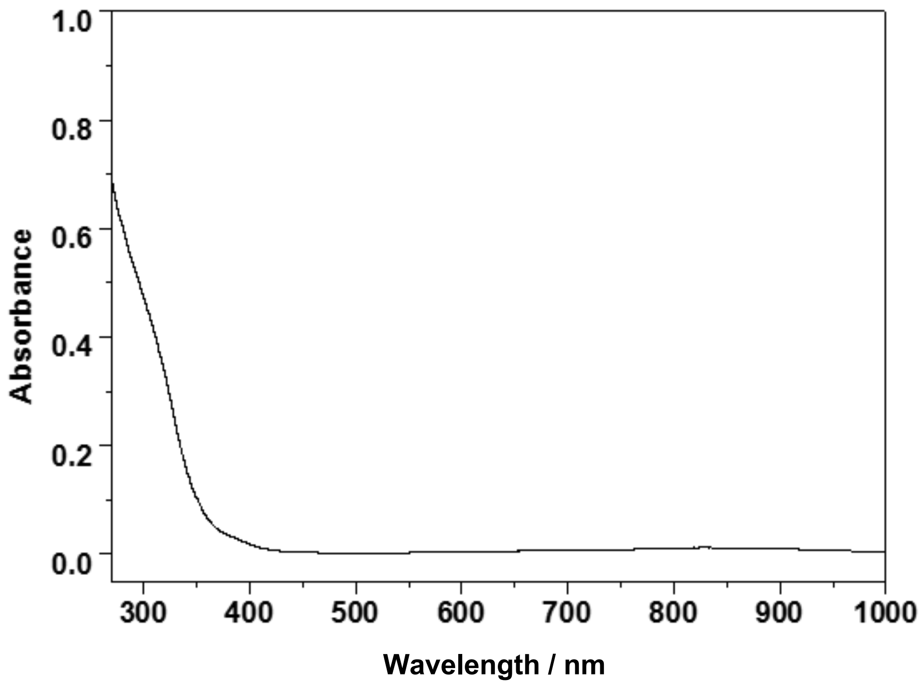

Figure S13. UV-Vis spectrum of **1** (2 μM in DMF).

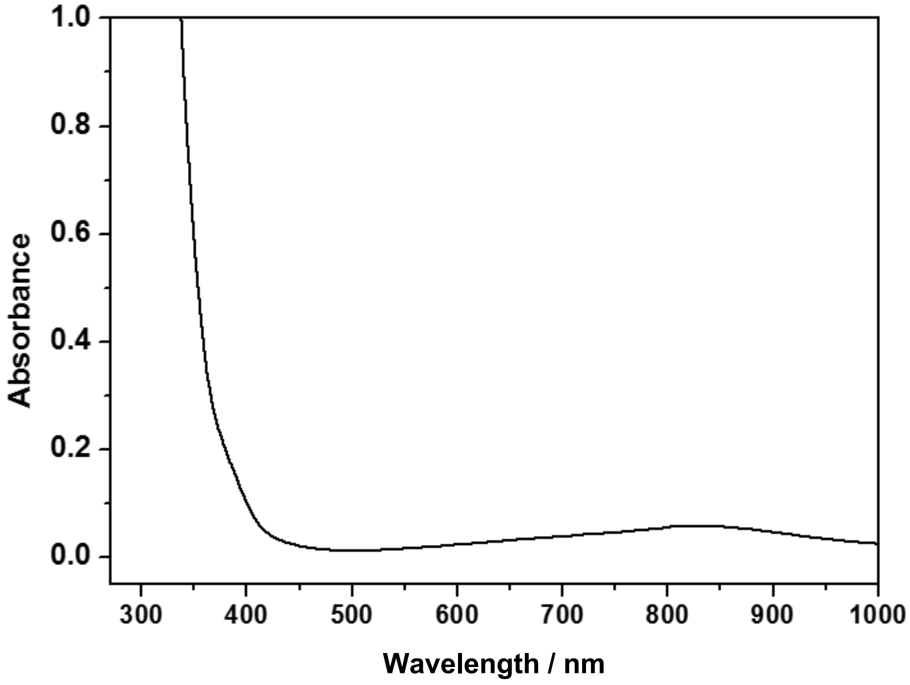

Figure S14. UV-Vis spectrum of reduced **1** (**1_{red}**) (19.5μM in DMF).

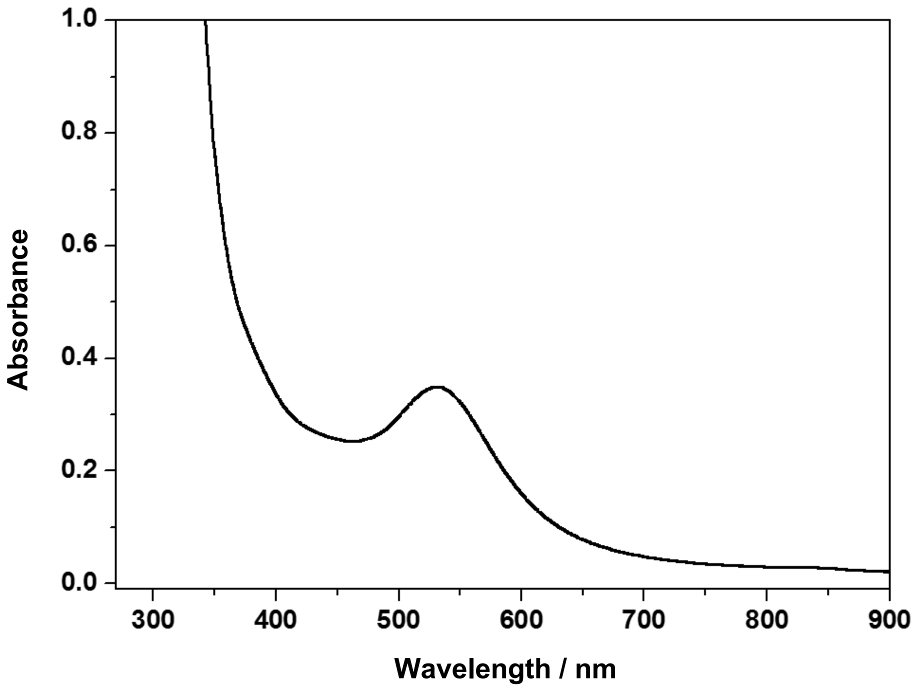

Figure S15. UV-Vis spectrum of **NP-1** in DMF. Gold surface plasmon resonance band λ_{max} = 531 nm.

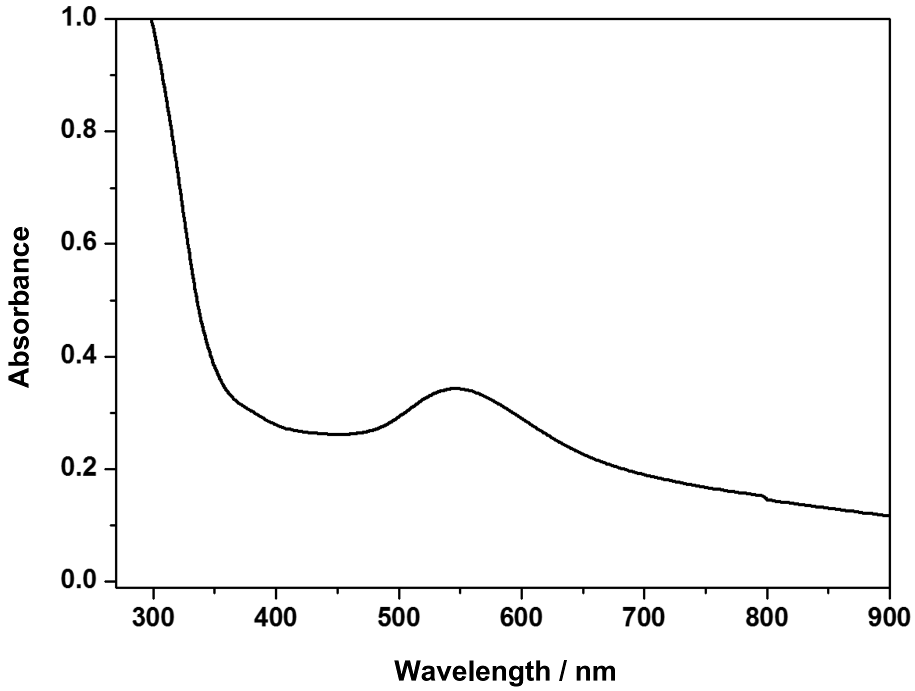

Figure S16. UV-Vis spectrum of **NP-P_2W_{18}** in DMF. Gold surface plasmon resonance band λ_{max} = 543 nm.

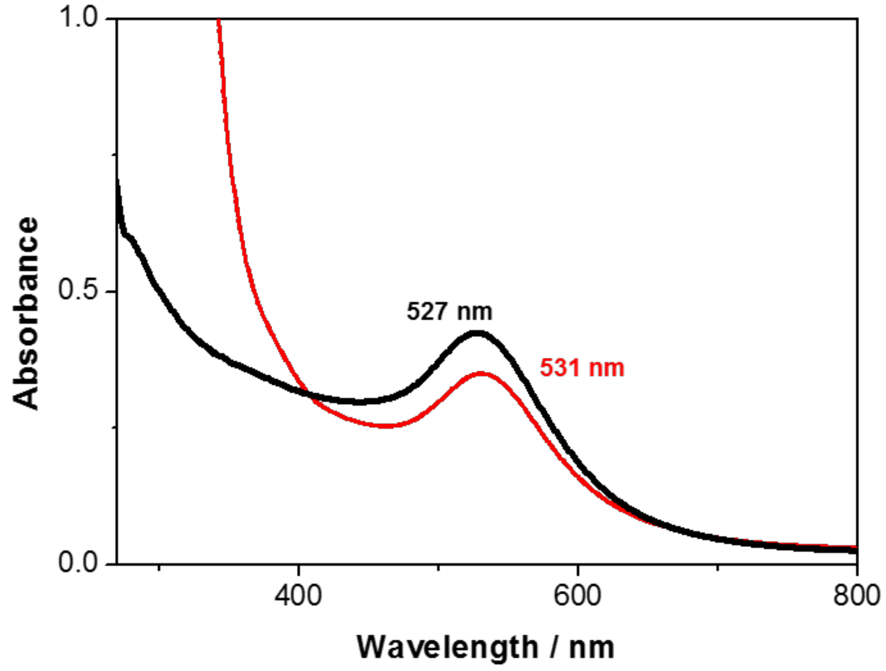

Figure S17. Comparative UV-Vis spectrum of **NP-1** (red trace) and **AuNPs@citrate** (black trace), both in DMF.

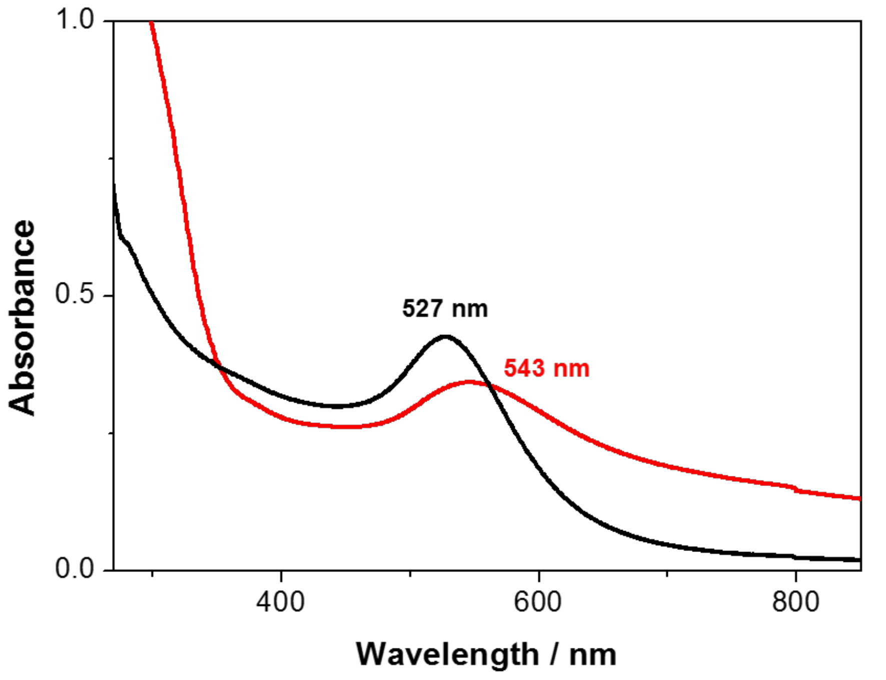

Figure S18. Comparative UV-Vis spectrum of **NP-P_2W_{18}** (red trace) and **AuNPs@citrate** (black trace), both in DMF.

6. ELECTROCHEMISTRY

All voltammetry measurements were performed on a CHI600e (CH Instruments) workstation.

Cyclic voltammetry measurements. A three-component system consisting of a glassy carbon working electrode (d = 1.6 mm), a Ag-wire reference electrode and Pt wire counter electrode were used. All potentials are reported *vs.* ferrocene as an internal standard. Experiments were performed under a positive pressure of N_2 at r.t. and in dry DMF solutions 0.1 M of tetrabutylammoniumhexa-fluorophosphate (NBu_4PF_6) was used as supporting electrolyte.

Table S1. Observed Mid-Point Redox Potentials (E).[a],[b]

	E_1/V (ΔE_1)	E_2/V (ΔE_2)
**$K_6[P_2W_{17}O_{61}(PO_2C_{17}H_{26}SH)_2]$, 1**[c]	-0.74 (0.07)	-1.12 (0.10)
**NP-1**[d]	-0.76 (0.09)	-1.15 (0.14)
**$K_6P_2W_{18}O_{62}$, {P_2W_{18}}** [c]	-1.17 (0.07)	-1.46 (0.08)
**NP-P_2W_{18}**[d]	-1.17 (0.07)	-1.46 (0.07)

[a] CV measurements using a glassy carbon working electrode of d = 1.6 mm. [b] All potential reported *vs* Fc/Fc^+. $E = (E_{p,c} + E_{p,a})/2$, where $E_{p,c}$ = peak cathodic potential and $E_{p,a}$ = peak anodic potential. $\Delta E = E_{p,c} - E_{p,a}$. [c] [POM] = 0.125 mM and scan rate of 10 mV s-1.[d] 4 mg of NPs in 2.5 mL of DMF and scan rate of 10 mV s-1.

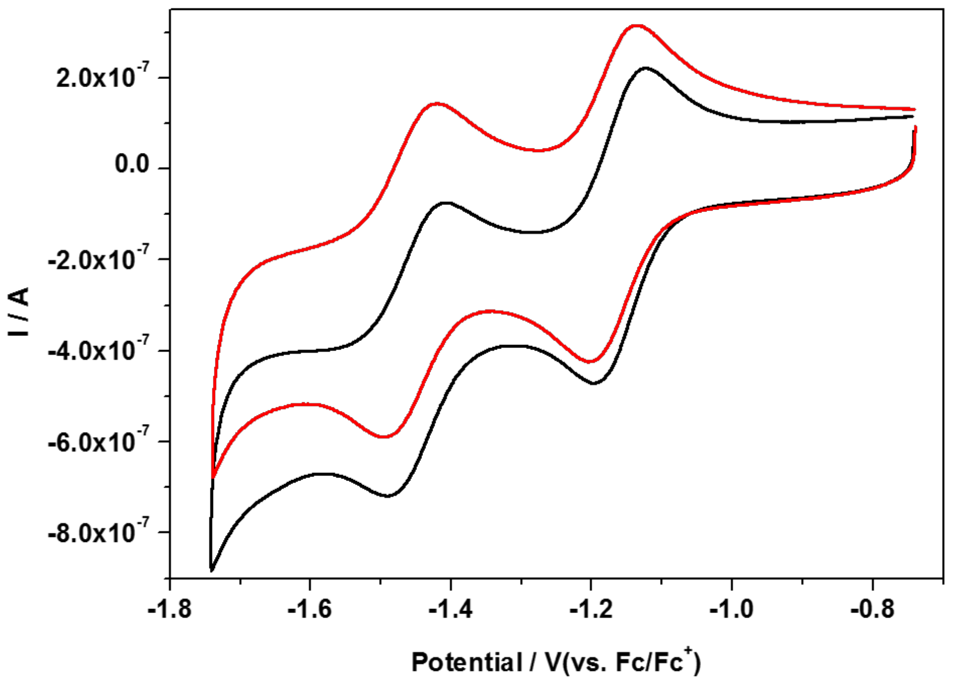

Figure S19. Comparative cyclic voltammogram of **NP-P_2W_{18}** (4 mg in 2.5 mL DMF, red trace) and **$K_6P_2W_{18}O_{62}$, {P_2W_{18}}** (125μM, black trace). GC working electrode (d = 1.6 mm). Scan rate of 10 mV s^{-1}.

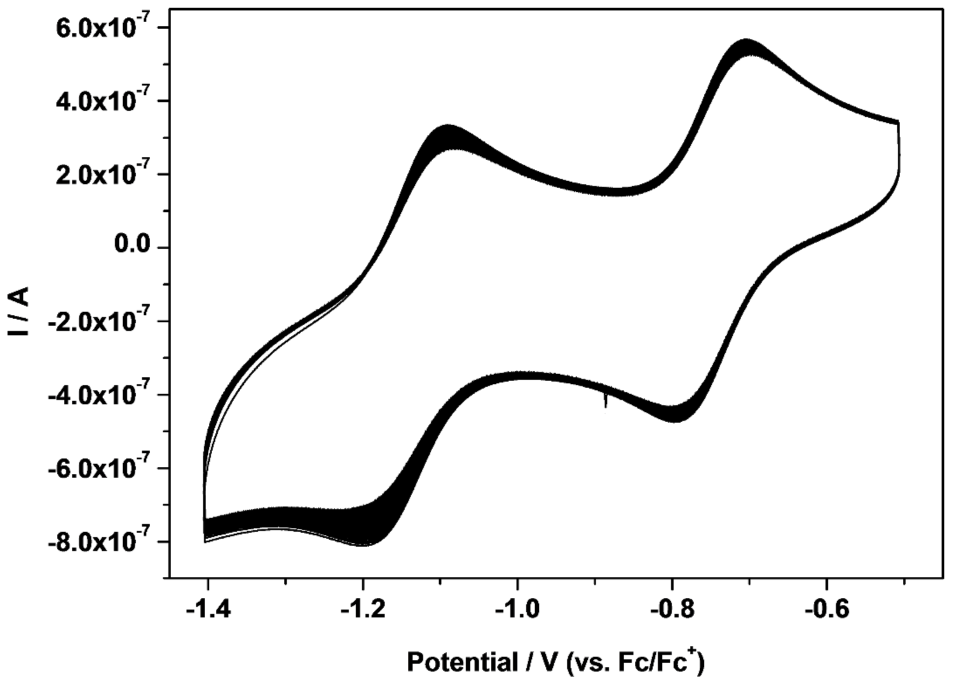

Figure S20. Consecutive cyclic voltammetry of **NP-1** (4 mg in 2.5 mL DMF, 50 scans), resulting in a 9.8 % reduction in peak intensity of the first reduction wave and 12.5 % in the intensity of the second reduction wave. Scan rate of 20 mV s-1.

Ultramicroelectrode cyclic voltammetry measurements. A three-component system consisting of a Pt working microelectrode (d = 25μm), a Ag-wire reference electrode and Pt wire counter electrode was used. All potentials are reported against ferrocene as an internal reference. Experiments were performed under N_2 atmosphere at r.t. and in dry DMF solutions 0.1 M of tetrabutylammoniumhexafluorophosphate (NBu_4PF_6) was employed as supporting electrolyte. All measurements were performed at a scan rate of 10 mV s^{-1}.

Table 2. Observed Mid-Point Redox Potentials (*E*).[a], [b]

	E / V
**$K_6[P_2W_{17}O_{57}(C_{17}H_{27}O_4PS)_2]$, 1**	-1.45
**NP-1**	-1.46

[a] CV measurements using a Pt working electrode of d = 25 μm.[b] All potential reported *vs* Fc/Fc^+. $E = (E_{p,c} + E_{p,a})/2$, where $E_{p,c}$ = peak cathodic potential and $E_{p,a}$ = peak anodic potential.

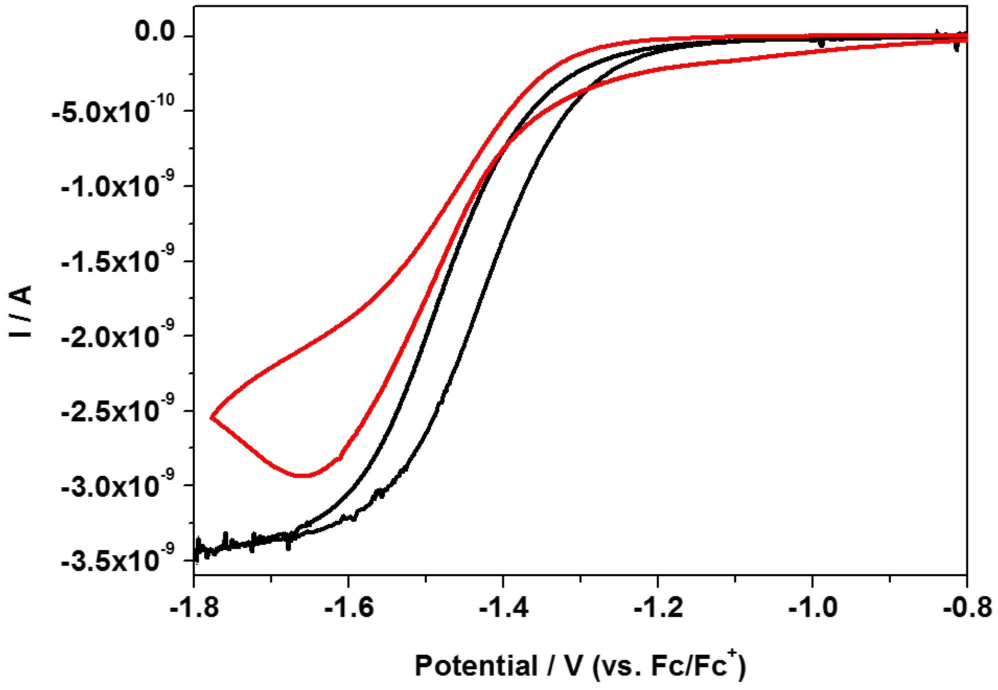

Figure S21. Comparative ultramicroelectrode cyclic voltammogram of **NP-1** (4 mg in 2.5 mL DMF, red trace) and **1** (125 μM, black trace). Scan rate = 10 mV s^{-1}.

7. TEM IMAGES

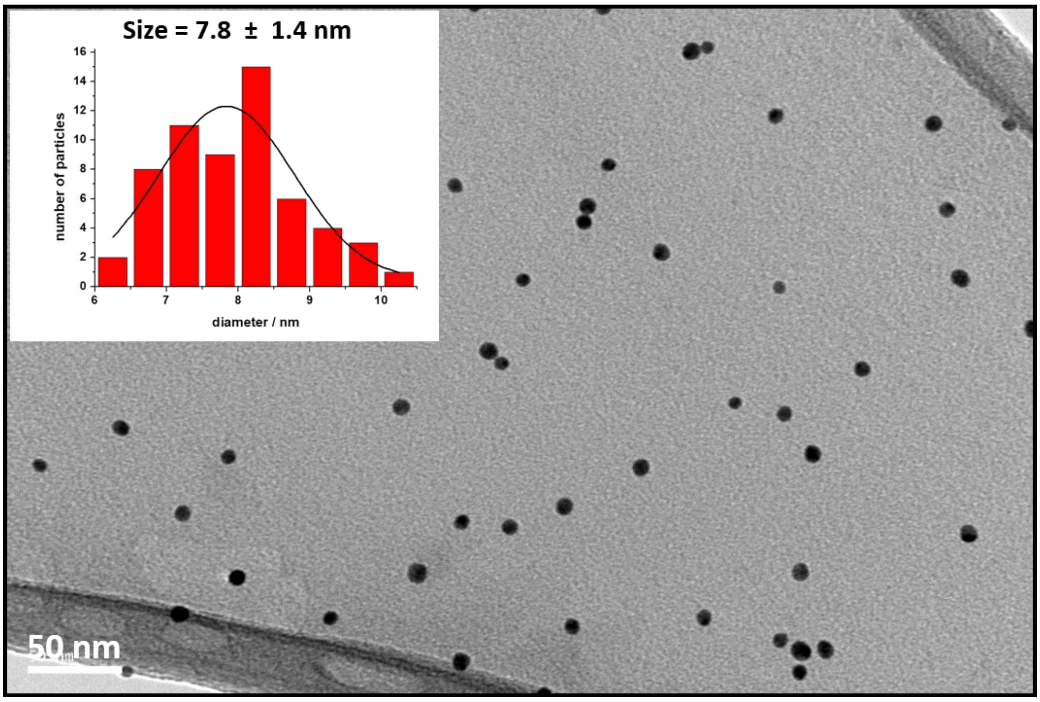

Figure S22. TEM micrograph of commercially sourced ‘10nm’ Au@citrate nanoparticles used as the precursor to the POM-stabilised nanocomposites. Size distributions (from TEM) shown inset.

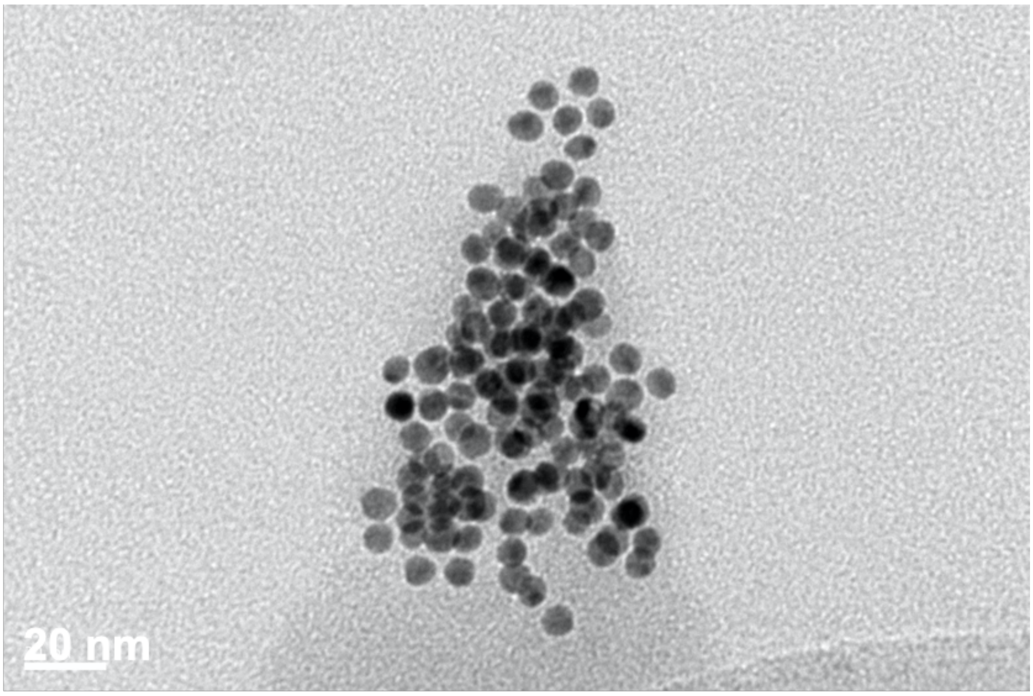

Figure S23. TEM micrograph of **NP-P_2W_{18}**.

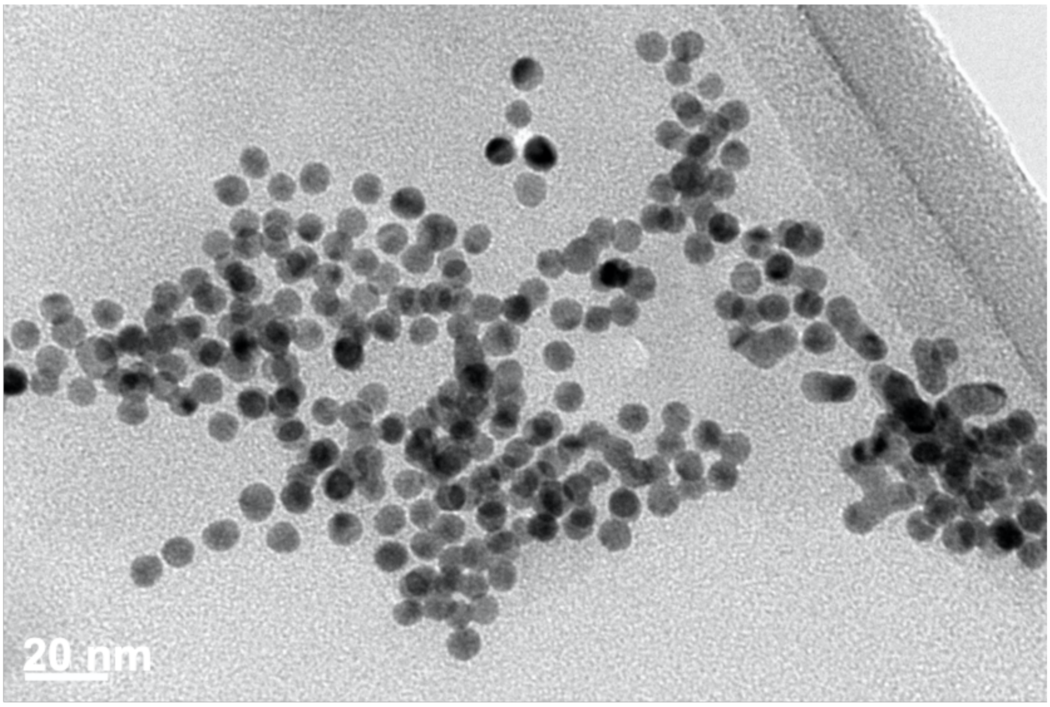

Figure S24. TEM micrograph of **NP-P_2W_{18}**.

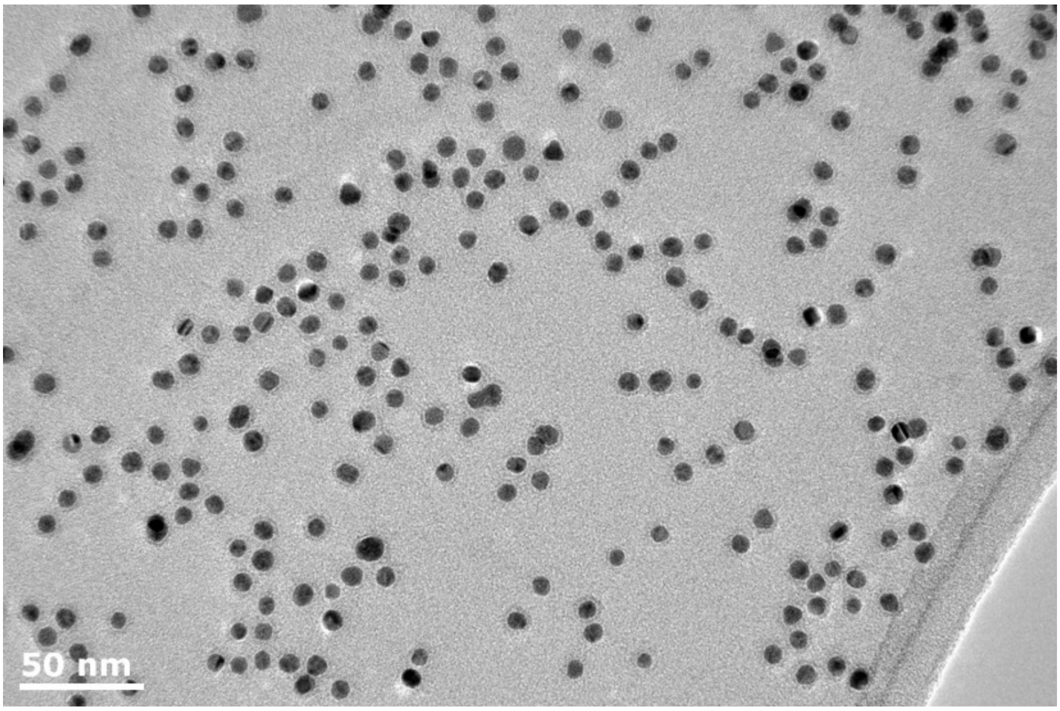

Figure S25. TEM micrograph of **NP-1.**

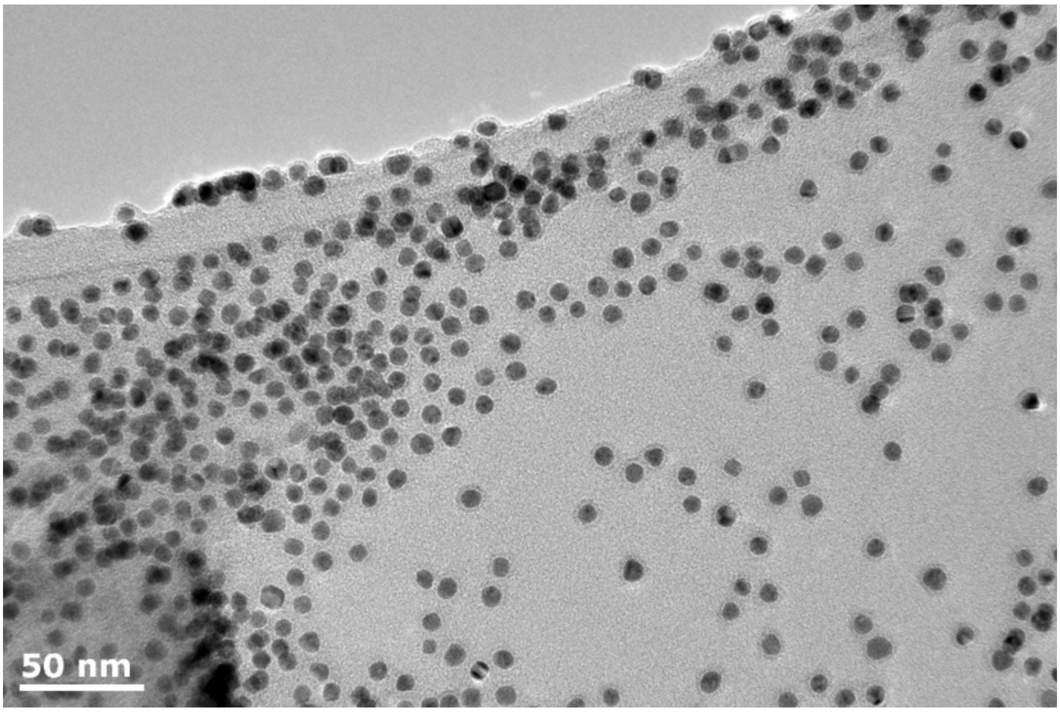

Figure S26. TEM micrograph of **NP-1**.

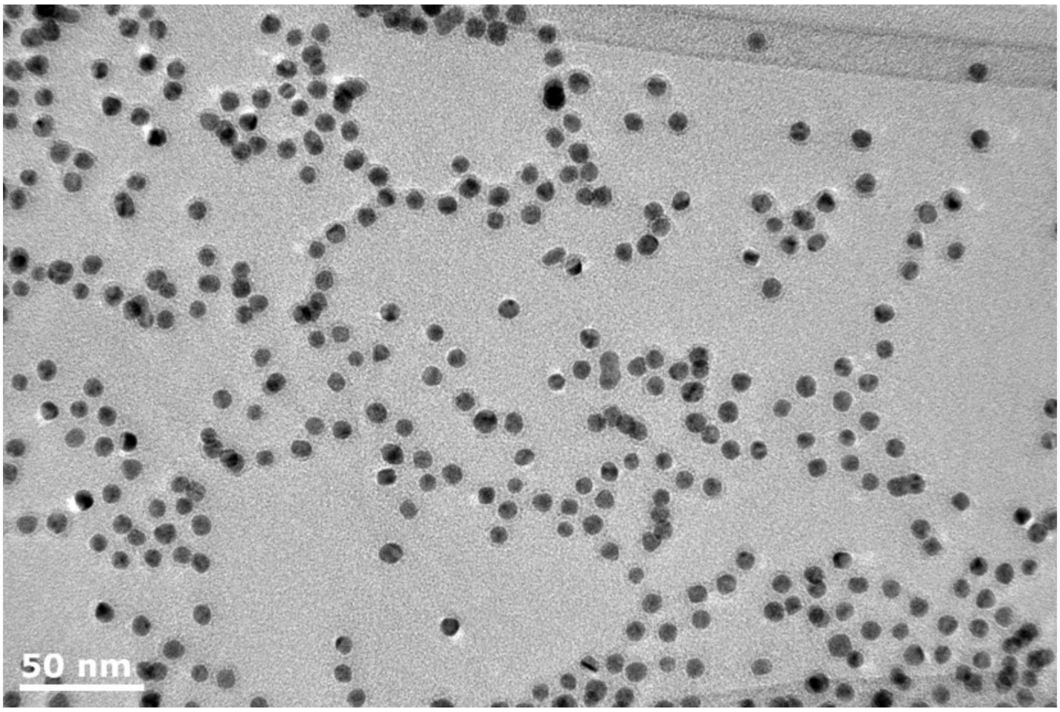

Figure S27. TEM micrograph of **NP-1**.

8. CALCULATION OF AVERAGE NUMBER OF POMs PER NANOPARTICLE

If we assume spherical and uniform nanoparticles, the average number of gold atoms per nanoparticles (N) can be calculated by the following equation: [5]

$$N = \frac{\pi}{6}\frac{\rho D^3}{M} = 14105$$

where ρ is the density of fcc gold (19.3 g/cm^3), M is the atomic weight of gold (197 g/mol) and D is the average core diameter of AuNPs in nm obtained by HR TEM.

With this data in hand, the average number of POM ligands per nanoparticle may be calculated by the used of ICP-OES analysis and by UV-vis spectroscopy (colorimetry).

8.1 ICP-OES ANALYSIS

The Gold to Tungsten ratio for **NP-1** was determined by ICP-OES, and is calculated to be in the proportion of 34:1 gold atoms to POM clusters (each POM containing 17 tungsten atoms). If the average number of gold atoms per nanoparticles (N) is 14105 and the Gold:POM ratio is 34:1, the average number of POMs per nanoparticles can be estimated as 414.

8.2 COLORIMETRY

The average number of POMs per nanoparticle can be also calculated by UV-vis spectroscopy (colorimetry). In this experiment, the reduced hybrid POM (**1**$_{\mathbf{red}}$) is used as analyte, with a strong characteristic absorbance at 820 nm. A calibration plot (Figures S28 and S29) were prepared using different solutions with concentrations of **1** between 1-40 μM. Solutions were irradiated for 45 min with a solar simulator (200 W) fitted with a 395 nm cut-off filter (Figure S28). Similarly, a solution of 0.25 mg of **NP-1** in 3 mL DMF was was irradiated for 45 min under the same conditions and its UV-vis spectrum was also recorded (black trace in Figure S28). The POM concentration in this sample was obtained by comparison to the calibration profile shown in Figure S29:

Abs$_{(\mathbf{NP\text{-}1})}$ = 0.0548 → [POMs] = 8.4 μM → Au-to-POM ratio is 25:1 → 564

Here, if the average number of gold atoms per nanoparticles (N) is 14105 and the gold-to-POMs ratio is 25:1, the average number of POMs per nanoparticles can be estimated as 564.

---

[5] X. Liu, M. Atwater, J. Wang, Q. Huo, *Colloids Surf. B: Biointerfaces* **2007**, *58*, 3–7.

We attribute the difference between the Gold-to-POM ratio and the number of POMs per nanoparticle calculated by both methods to be due to systematic error in the colorimetry analysis, since the Au plasmon resonance band and POMs intervalence charge transfer bands somewhat overlap in the UV-vis spectrum of **NP-1**.

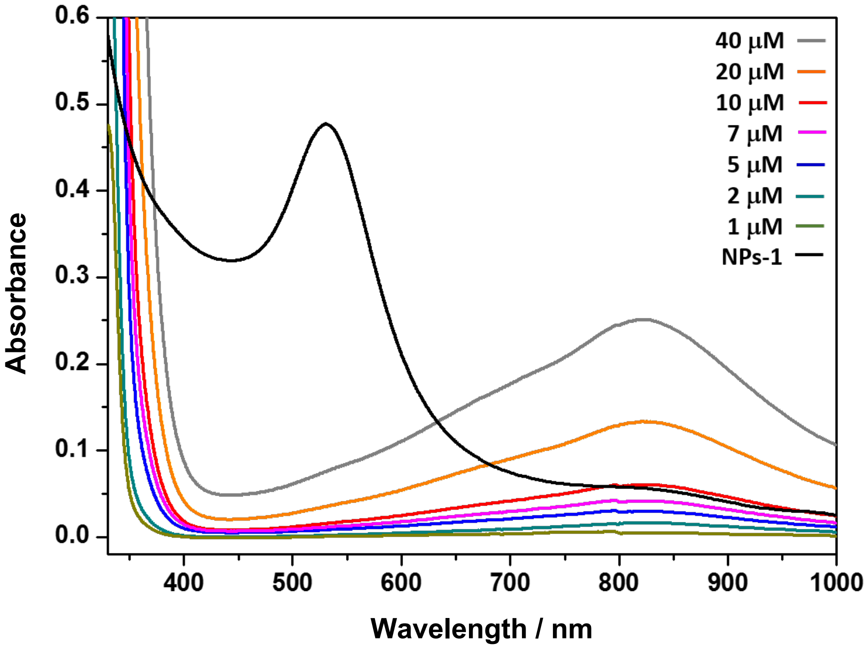

Figure S28. UV-Vis spectra in DMF of **1** at different concentrations (1-40 μM) and **NP-1** (0.25 mg in 3 mL) irradiated with a solar simulator fitted with a 395 nm cut off filters during 45 min.

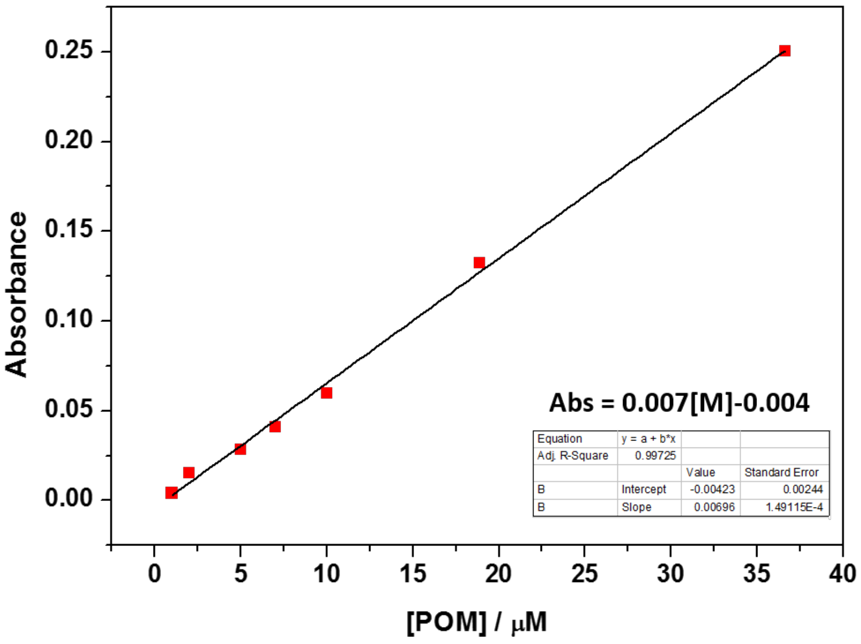

Figure S29. Plot of Absorbance versus concentration for **1**.

9. STABILTY STUDIES OF NP-1 AND NP-P_2W_{18}

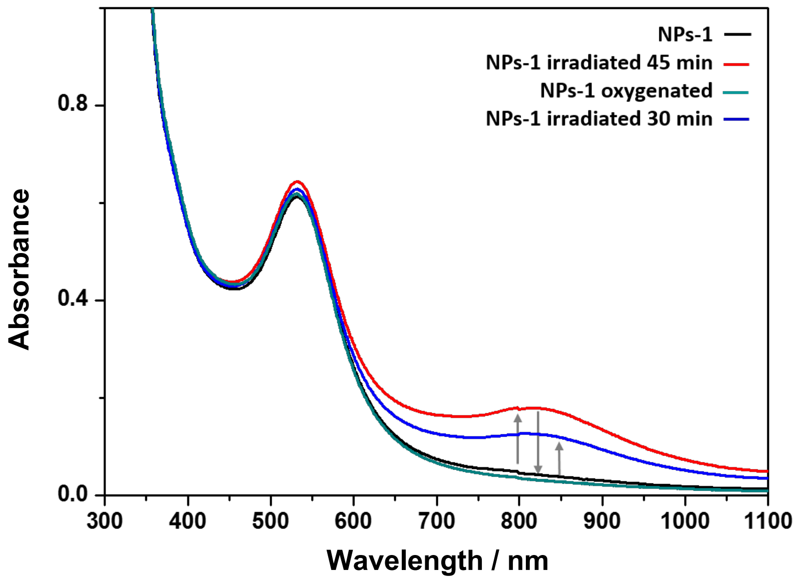

Figure S30. UV-Vis spectra of **NP1** in DMF (black trace); **NP1** irradiated with a solar simulator fitted with a 395 nm cut-off filter for 45 min (red trace); **NP1** re-oxidized by bubbling with O_2 for 1h (green trace), and; **NP1** re-irradiated for 30 min under the same conditions (blue trace).

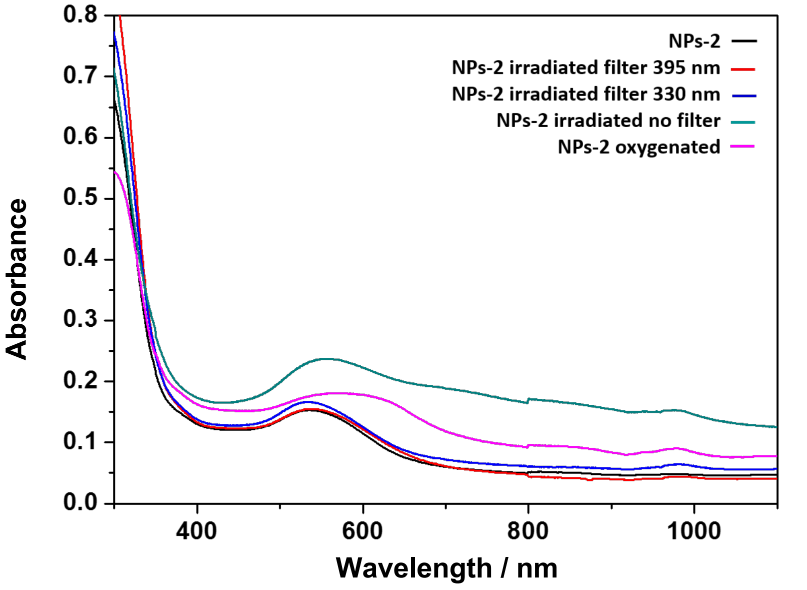

Figure S31. UV-Vis spectra in DMF of **NP-P_2W_{18}** (black trace); **NP-P_2W_{18}** irradiated with a solar simulator fitted with a 395 nm cut off filter for 45 mins (red trace); **NP-P_2W_{18}** irradiated with a solar simulator fitted with a 330 nm cut off filter for 45 mins (blue trace); **NP-P_2W_{18}** irradiated without cut-off filters during 45 mins (green trace) and photoreduced **NP-P_2W_{18}** (green trace) oxidized by bubbling O_2 for 60 mins (pink trace). In comparison to Figure 3 in the manuscript, this clearly shows both the markedly lower photoreactivity and instability of **NP-P_2W_{18}** in comparison to NP-1 (shown by the shift in the gold surface plasmon upon successful reduction of the POM).

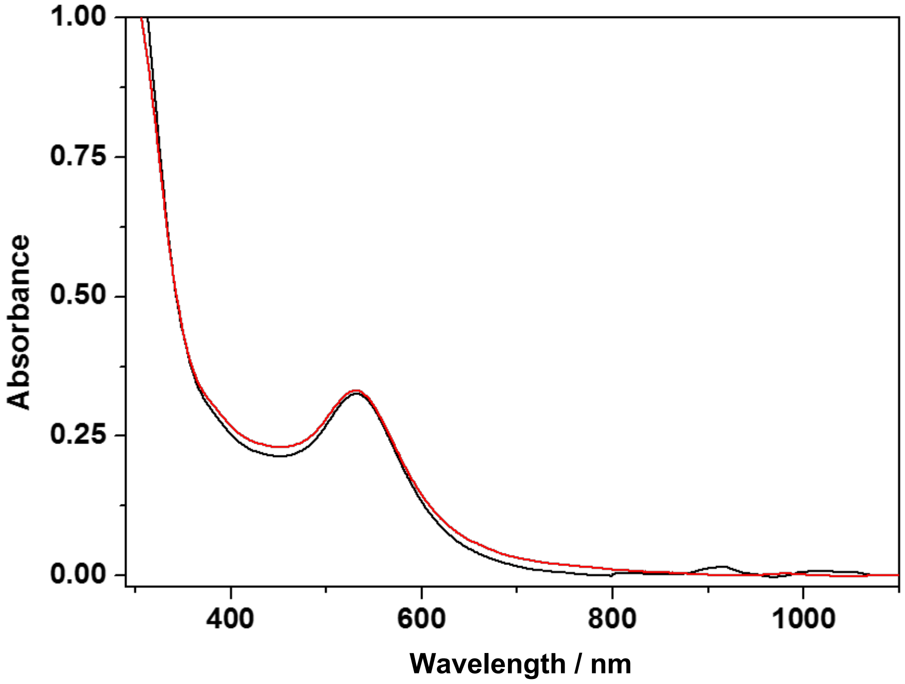

Figure S32. UV-Vis spectra in DMF of **NP-1** at room temp. (black trace) and **NP-1** after heating to 80 ºC for 24 h (red trace). The gold surface plasmon resonance (Au-SPR) band appears at 531 nm in both cases.

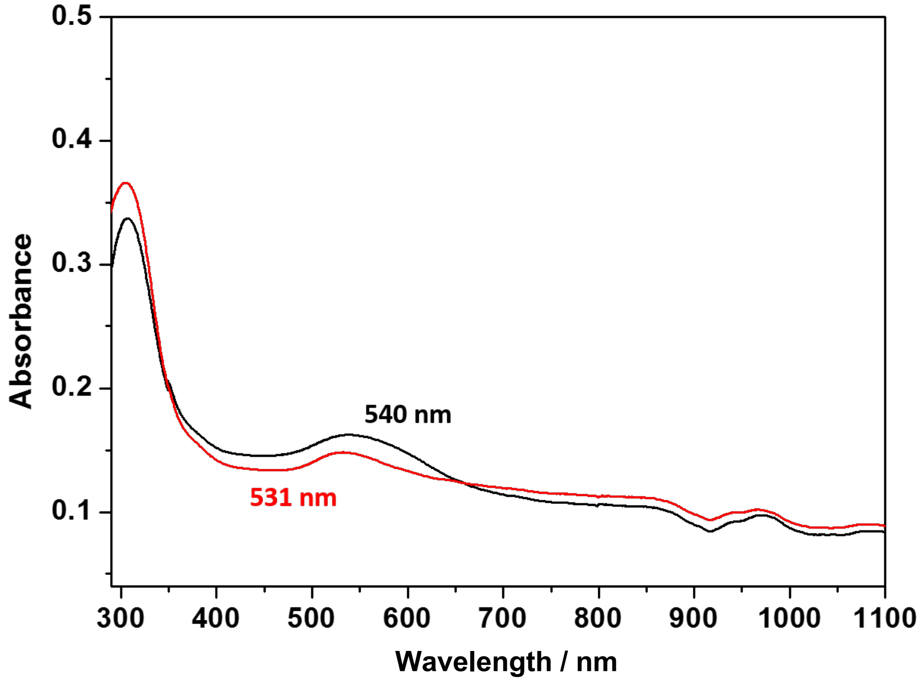

Figure S33. UV-Vis spectra in DMF of **NP-P_2W_{18}** at room temp. (Au-SPR band at 540 nm, black trace) and **NP-P_2W_{18}** after heating to 80 ºC for 24 h with gold Plasmon resonance band shifted to 531 nm (black trace), indicating decomposition of the composite.

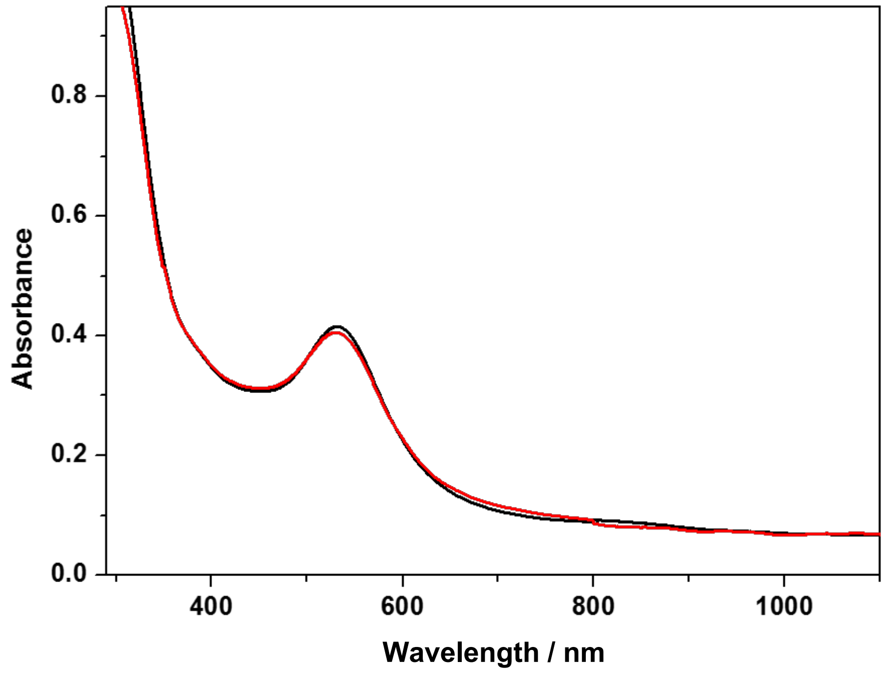

Figure S34. UV-Vis spectra in DMF of **NP-1** at room temp. (black trace) and **NP-1** after treatment with 0.5 % H_2O_2 for 24 h (red trace). Au-SPR band appears at 531 nm in both cases.

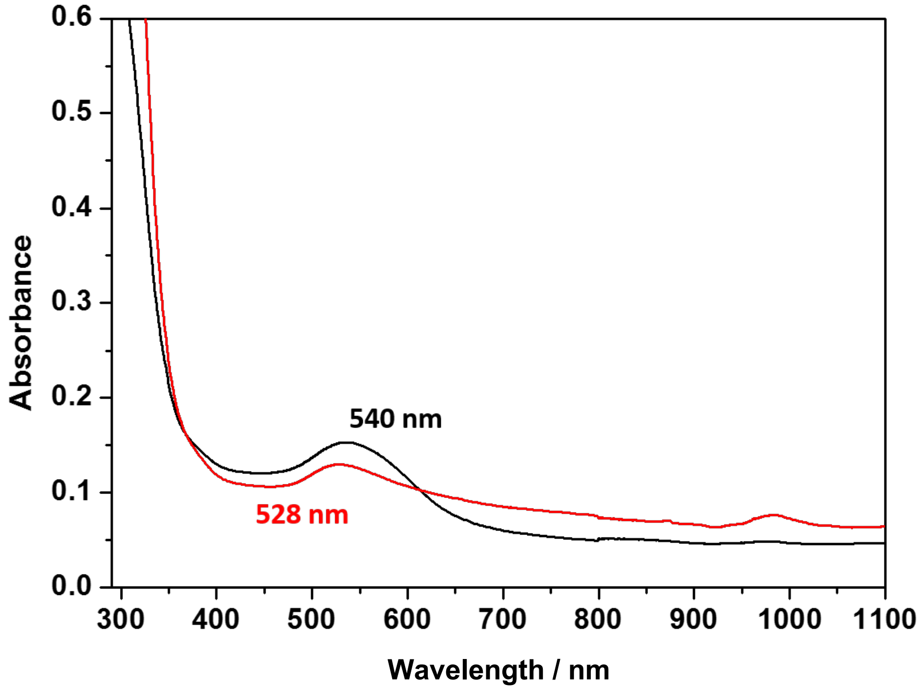

Figure S35. UV-Vis spectra in DMF of **NP-P_2W_{18}** at room temp. with Au-SPR band at 540 nm (black trace) and **NP-P_2W_{18}** after treatment with 0.5 % H_2O_2 for 24 h with Au-SPR band shifted to 528 nm (red trace), indicating decomposition.

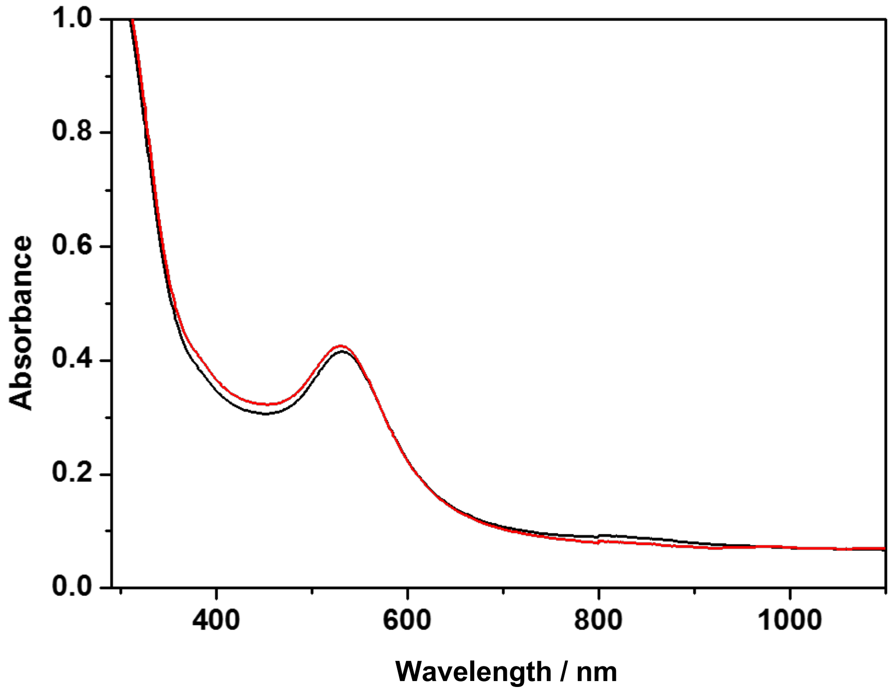

Figure S36. UV-Vis spectra in DMF of **NP-1** at room temp. (black trace) and **NP-1** after 24h at 37 ºC in pH 7 buffer (red trace). Au-SPR band at 631 nm in both cases.

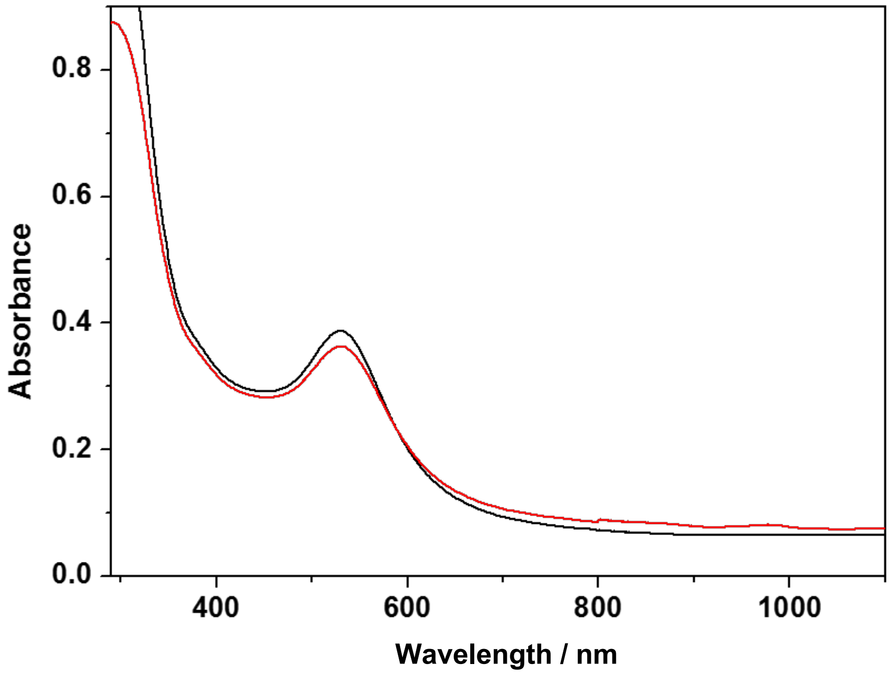

Figure S37. UV-Vis spectra in DMF of **NP-1** at room temp. (black trace) and **NP-1** after 24h at 37 ºC in pH 5 buffer (red trace). Au-SPR band at 631 nm in both cases.

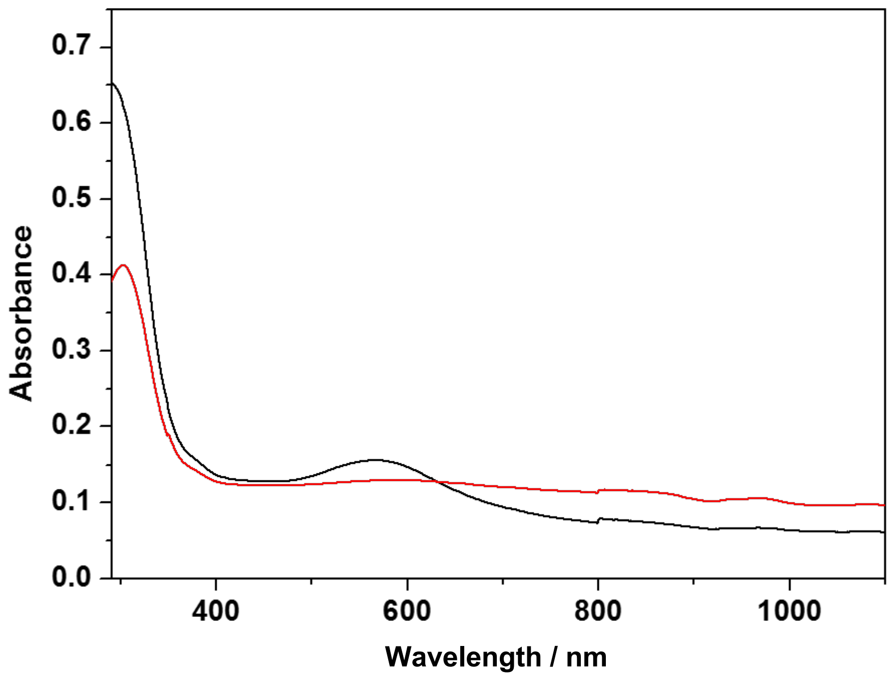

Figure S38. UV-Vis spectra in DMF of **NP-P_2W_{18}** at room temp. (black trace) and **NP-P_2W_{18}** after 24h at 37 ºC in pH 5 buffer (red trace). Loss of a clear Au-SPR band indicates decomposition of the composite.
